# Atypicalities in sleep and semantic consolidation in autism

**DOI:** 10.1111/desc.12906

**Published:** 2019-10-20

**Authors:** Fay E. Fletcher, Victoria Knowland, Sarah Walker, M. Gareth Gaskell, Courtenay Norbury, Lisa M. Henderson

**Affiliations:** ^1^ Department of Psychology University of York York UK; ^2^ Division of Psychology and language Sciences UCL London UK

**Keywords:** autism spectrum disorder, children, memory consolidation, sleep, vocabulary, word learning

## Abstract

Sleep is known to support the neocortical consolidation of declarative memory, including the acquisition of new language. Autism spectrum disorder (ASD) is often characterized by both sleep and language learning difficulties, but few studies have explored a potential connection between the two. Here, 54 children with and without ASD (matched on age, nonverbal ability and vocabulary) were taught nine rare animal names (e.g., pipa). Memory was assessed via definitions, naming and speeded semantic decision tasks immediately after learning (pre‐sleep), the next day (post‐sleep, with a night of polysomnography between pre‐ and post‐sleep tests) and roughly 1 month later (follow‐up). Both groups showed comparable performance at pre‐test and similar levels of overnight change on all tasks; but at follow‐up children with ASD showed significantly greater forgetting of the unique features of the new animals (e.g., pipa is a *flat* frog). Children with ASD had significantly lower central non‐rapid eye movement (NREM) sigma power. Associations between spindle properties and overnight changes in speeded semantic decisions differed by group. For the TD group, spindle duration predicted overnight changes in responses to novel animals but not familiar animals, reinforcing a role for sleep in the stabilization of new semantic knowledge. For the ASD group, sigma power and spindle duration were associated with improvements in responses to novel and particularly familiar animals, perhaps reflecting more general sleep‐associated improvements in task performance. Plausibly, microstructural sleep atypicalities in children with ASD and differences in how information is prioritized for consolidation may lead to cumulative consolidation difficulties, compromising the quality of newly formed semantic representations in long‐term memory.

## Graphical abstract

This polysomnography study examined the sleep‐associated consolidation of words for real but rare animals, in children with and without autism. Children with autism showed increased forgetting of novel features (e.g., an asp is a *hairy* caterpillar), relative to typical peers, one month after exposure. They also showed reduced sigma power (within the sleep spindle frequency range), and showed more general associations between overnight changes in task performance (for both novel and particularly familiar words) and sleep spindle parameters, in stark contrast to typical peers who showed selective associations between spindles and overnight changes in novel memory traces.

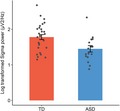

**Research Highlights**

- Initial learning and overnight consolidation of the names and meanings of novel animals were comparable in children with autism and typical peers.
- A month after learning, children with autism were more likely to forget the unique features of the new animals than typical peers.
- Children with autism showed lower sigma power on the night after learning than typical peers.
- Associations between spindle parameters and overnight changes in semantic decision speed were specific to novel animals in the typical (but not the autism) group.

## 1 INTRODUCTION

Sleep is pivotal to brain plasticity and learning and plays a key role in facilitating memory consolidation, by which new and initially weak memories become strengthened and resistant to interference (e.g., Born, 2010; Rasch & Born, 2013). The benefits of sleep for declarative memory (e.g., for facts) are well‐established in adults (Gais, Lucas, & Born, 2006; Plihal & Born, 1997; Tucker et al., 2006), with comparable or enhanced benefits in children (Kurdziel, Duclos, & Spencer, 2013; Wilhelm, Diekelmann, & Born, 2008; Wilhelm et al., 2013). For instance, improvements in memory for novel word meanings after sleep compared to wake have been reported in infants (Friedrich, Wilhelm, Born, & Friederici, 2015), children (Ashworth, Hill, Karmiloff‐Smith, & Dimitriou, 2014; Williams & Horst, 2014) and adults (Kurdziel & Spencer, 2016). Such benefits can be explained by the complementary learning systems (CLS) framework (McClelland, McNaughton & O’Reilly, 1995), which proposes that newly formed hippocampal memory traces are reactivated during sleep to facilitate consolidation in neocortical memory circuits. However, an effective model of consolidation must also be able to account for individual differences. Indeed, findings of different or reduced benefits of sleep are emerging for children with ADHD (Prehn‐Kristensen et al., 2013), dyslexia (Smith et al., 2018), Williams syndrome, Down syndrome (Ashworth, Hill, Karmiloff‐Smith, & Dimitriou, 2017; Spanò, Gómez, Demara, Cowen, & Edgin, 2017) and autism spectrum disorder (ASD; Maski et al., 2015). Studies of neurodevelopmental disorders have the potential, therefore, to offer valuable theoretical insight into individual differences in consolidation processes (see Smith & Henderson, 2016, for discussion of this in the context of dyslexia).

Importantly, dialogue between the hippocampus and neocortex is thought to be orchestrated by sleep spindles: distinct trains oduring infant sleep. Nature Communicationsf sinusoidal EEG activity at 10–15 Hz, lasting approximately 0.5–3 s (Rasch & Born, 2013). Sleep spindles are thalamically generated during non‐rapid eye movement (NREM) sleep, and are proposed to support consolidation via their temporal synchrony with hippocampal sharp‐wave ripples and neocortical slow oscillations (Antony, Schönauer, Staresina, & Cairney, 2018; Diekelmann & Born, 2010; Genzel et al., 2017; Latchoumane, Ngo, Born, & Shin, 2017; Staresina et al., 2015). It has been hypothesized that spindle‐orchestrated ‘replaying’ patterns of hippocampal and neocortical activity following learning are key to the ‘whole‐brain reorganization’ required for cellular consolidation across distributed neocortical connections (i.e., systems consolidation; Genzel et al., 2017; see Runyan, Moore, & Dash, 2019, for a review). Sleep spindles have been shown to occur more frequently after learning, and have been associated with synaptic plasticity and improved retention (Muller et al., 2016; Rosanova & Ulrich, 2005). Within the domain of word learning, it has been demonstrated that overnight improvements in lexical stabilization and integration are associated with spindle characteristics measured via polysomnography in adults (Tamminen, Payne, Stickgold, Wamsley, & Gaskell, 2010; Weighall, Henderson, Barr, Cairney, & Gaskell, 2017) and children (Smith et al., 2018). Sleep spindle density has also been linked to the integration of new knowledge into a previously learned memory schema, and with increasing independence from hippocampus during recall the subsequent day (Hennies, Ralph, Kempkes, Cousins, & Lewis, 2016).

While the role of sleep is well‐established in phonological aspects of word learning (see James, Gaskell, Weighall & Henderson, 2017), there is less evidence relating to semantic aspects of vocabulary consolidation. Tham, Lindsay, and Gaskell (2015) provided evidence for a role for sleep in consolidating novel form‐meaning mappings. Adult participants learnt Malay translations for nine English animal names and were later tested using a size judgement paradigm, after a period of either sleep or wake. Participants were presented with two English or Malay animal names written on screen and had to decide which animal was larger. Font size congruent (BEE‐COW) and incongruent (BEE‐COW) trials were included, such that if meaning was automatically retrieved upon presentation of the written words, then response times (RTs) should be faster for congruent trials (Rubinsten & Henik, 2002). Two key findings emerged. First, evidencing *semantic stabilization*, overall task RTs were quicker in the sleep than the wake group for Malay, but not English trials, regardless of congruency. This pattern suggested that sleep led to more efficient semantic processing of the newly learned items. Additionally, the sleep group demonstrated a size congruency effect for the Malay trials (signalling automatic semantic retrieval, owing to *semantic integration*). However, this effect was weak and only evident in trials for which there was a larger size difference between animals. Nevertheless, larger congruency effects for these trials were associated with greater spindle density in the sleep group. The authors argued that this supports a systems consolidation account of declarative learning, with sleep playing an active role in the integration of novel semantic information into existing networks.

These findings resonate with a recent infant study, in which state‐dependent changes in spindle density predicted generalization of novel object labels 1 day after learning (Friedrich, Mölle, Friederici, & Born, 2018). Furthermore, a developmental MEG study in children aged 8–12 years found that activity in inferior frontal gyri and medial prefrontal cortex was associated with recall of novel object associations (i.e., semantic learning) following sleep but not wake; whereas the wake group showed significantly greater hippocampal activation (Urbain et al., 2016). Thus, there is emerging evidence that sleep, particularly spindle activity, supports the consolidation of new semantic material in development, as well as in adulthood.

The above findings have potential implications for individuals with neurodevelopmental disorders characterized by atypical sleep. ASD is a pervasive neurodevelopmental disorder, with prevalence between 1/34 and 1/76 (Baio et al., 2018). Sleep disorders are claimed to be present in up to 80% of ASD children, most often characterized by longer sleep onset latency and reduced sleep efficiency (Diaz‐Roman, Shang, Delorme, Zhang, Delorme, Beggiato, & Cortese, 2018; Fletcher et al., 2017; Souders et al., 2009). However, few studies have utilized polysomnography to objectively explore sleep in children with ASD. There is some evidence to suggest that sleep spindles may differ in ASD (Gruber & Wise, 2016), with reduced N2 central spindle density (spindles per minute) in adults (Godbout, Bergeron, Limoges, Stip, & Mottron, 2000; Limoges, Mottron, Bolduc, Berthiaume, & Godbout, 2005). In a sample of 13 children with ASD, no differences in N2 central spindle density were observed compared with controls (Lambert et al., 2016; see also Maski et al., 2015), but there was significantly reduced central spindle duration and central sigma power in the same sample (i.e., power spectral density across the spindle frequency range, Tessier et al., 2015). It seems highly relevant, then, to explore the extent to which sleep supports memory consolidation in ASD.

A large proportion of children with ASD show early and persistent language delays, including impoverished vocabulary knowledge (Hudry et al., 2010; Hus, Pickles, Cook, Risi, & Lord, 2007; Tager‐Flusberg, Paul, & Lord, 2005). Many studies have examined vocabulary learning in ASD and shown strengths in the initial mapping of a new word to referent when social cues are salient (de Marchena, Eigsti, Worek, Ono, & Snedeker, 2011; Luyster & Lord, 2009; Parish‐Morris, Hennon, Hirsh‐Pasek, Golinkoff, & Tager‐Flusberg, 2007; Preissler, 2008; Swensen, Kelley, Fein, & Naigles, 2007). However, few studies have explored longer term retention. In one exception, Norbury, Griffiths, and Nation (2010) taught children with and without ASD (matched on receptive vocabulary) four novel object names and assessed memory (via definitions and naming tasks, to tap semantic and phonological knowledge respectively) immediately and a month later. Children with ASD showed poorer overall performance when asked to define the features of the novel objects than TD peers. Furthermore, whilst the typical peers showed further improvements in accuracy at the 1‐month follow‐up (+11%), the ASD group showed weaker feature recall (–5%). The ASD group outperformed TD peers at the immediate naming test, but this difference diminished at the 1‐month follow‐up because the TD peers (but not the ASD group) improved over time. Therefore, across tasks only the TD group showed results consistent with long‐term consolidation. The enhanced initial phonological performance in the ASD group immediately after learning aligns with Henderson, Powell, Gaskell, and Norbury (2014), where children with ASD showed evidence of immediate lexical integration of novel phonological forms, which was not maintained 24 hr later. Interestingly, explicit measures of phonological recall and word form recognition identified intact overnight consolidation mechanisms in children with ASD relative to their TD peers. Collectively then, previous data suggest that initial encoding of words may be spared or even enhanced in ASD, and overnight consolidation of word form information may be intact; however, difficulties may lie in longer term consolidation processes. Whilst it is plausible that such difficulties could be linked to atypical sleep architecture, this is yet to be established, particularly in relation to semantic aspects of word learning.

This study examined semantic aspects of rare word learning in school‐aged children with and without ASD, matched on age, vocabulary and nonverbal ability. We utilized polysomnography to investigate associations between key sleep parameters and behavioural changes in memory. Participants learnt the names of previously unfamiliar animals over a series of explicit training trials (e.g., reading aloud; word‐picture matching). Explicit memory was assessed via a naming task (to assess the accuracy and speed of phonological retrieval in response to the pictures) and a definitions task (to assess the depth of semantic knowledge). Furthermore, a size judgement task, based on Tham et al. (2015), assessed both *semantic stabilization* (speed of animal size judgement for novel and familiar animals) and *semantic integration* (size congruency for novel and familiar animals). The use of novel and familiar trials allowed us to examine whether children prioritized novel information for consolidation over already familiar information, similar to the adult findings from Tham et al. (2015). In addition to the familiar and novel trials used by Tham et al. (2015) we introduced ‘mixed’ trials, comprising one novel and one familiar animal. This formed a mid‐way condition between familiar and novel trials to explore the way in prior knowledge may scaffold semantic decision speed; whereby the difference between familiar and novel trials should be greater than the difference between familiar and mixed.

The following hypotheses were made: (a) The TD and ASD groups would demonstrate comparable performance immediately after learning when defining and naming the newly learned animals, but consolidation (particularly at a delayed follow‐up) may be stronger in TD than ASD groups (Henderson et al., 2014; Norbury et al., 2010); (b) For the size judgement task, RTs would reduce overnight for trials including novel animals (relative to trials containing already familiar animals, for which no sleep‐dependent consolidation would be required) and this consolidation benefit would be larger in TD than ASD, representing greater stabilization of novel semantic information in TD children. Further, if semantic integration occurred, then congruency effects (faster RTs for congruent than incongruent trials) would be evident after sleep in trials containing novel animals (particularly for trials with a large semantic distance, as in Tham et al., 2015); (c) children with ASD would show differences in sleep microstructure, including reduced NREM sleep duration, sigma power (i.e., power within the sleep spindle frequency range), spindle duration and/or spindle density; (d) Sleep spindle parameters would be associated with overnight changes in the semantic stabilization and integration of novel (but not familiar) animals.

## 2 METHODS

### 2.1 Participants

Children aged 8–12 years (*n* = 59), with and without autism, were recruited as part of the SleepSmart project at the University of York. The research team carried out the recruitment and selection of participants.

#### 2.1.1 Inclusion–exclusion criteria

Children were invited to participate following an initial screening interview administered over the phone to ensure they were (a) native monolingual English speakers, (b) had no diagnosis of epilepsy or genetic syndromes, (c) they had normal or corrected to normal vision and hearing and (d) they had no diagnoses of sleep disordered breathing.

Twenty‐five children were initially recruited for the ASD group. We excluded any children with diagnoses of co‐occurring conditions (i.e., leading to two children with ASD being excluded as a consequence of having dyslexia). Due to the high verbal demands of the experimental tasks, three children were also excluded due to scoring <75 on the British Picture Vocabulary Scale 3rd Edition (BPVS; Dunn, Dunn, & Styles, 2009). The remaining 20 children all met our inclusion criterion of either a formal diagnosis of autism (*n* 14) or an ongoing formal diagnostic assessment (*n* 6), which has an average duration for this age range of 3.5 years (Crane, Chester, Goddard, Henry, & Hill, 2016). In one large‐scale study, 70% of children referred for an autism diagnosis went on to receive a diagnosis, and for children without any co‐occurring conditions (as was the case for the present sample) this figure rose to 89% (Lo, Klopper, Barnes, & Williams, 2017). Importantly, all parents completed the Gilliam Autism Rating Scale (GARS) with all 20 children in the ASD group receiving GARS‐AI scores ≥71 (i.e., severity level 2 or 3). GARS‐AI scores (*M* = 97.70, *SD* = 15.49) did not differ significantly from the GARS normative sample of children with ASD (*t*(20) = 0.68, *p* = .50). An additional 34 additional children met inclusion criteria for the TD group: (a) not a sibling of a child with ASD, (b) GARS‐AI < 55 (i.e., below cut‐off for ‘probable’ parent‐report autism profiles), (c) no diagnosed psychological disorder.

#### 2.1.2 Group characteristics

As shown in Table 1, the two groups were matched for age, sex, receptive vocabulary (measured by the BPVS‐III) expressive vocabulary (measured by the Word Definitions verbal IQ subscale of the British Ability Scale 3rd Edition, Elliot & Smith, 2011) and nonverbal ability (measured by the Matrices nonverbal IQ subtest of the BAS‐3), with all *p*s > .05. Not surprisingly, the ASD group had significantly higher parent‐reported sleep problems (Children's Sleep Habits Questionnaire [CSHQ]; Owens, Spirito, & McGuinn, 2000), driven by higher scores on the sleep duration, night wakings, parasomnias and daytime sleepiness subscales. Notably, there were no significant differences between groups in the sleep apnoea subscale, and the mean scores for both groups were comparable to the normative TD sleep apnoea subscale (Owens et al., 2000, one sample *t* test *p* > .05), reinforcing our exclusion criteria pertaining to sleep disordered breathing. The group with autism was also characterized by higher parent‐reported internalizing and externalizing symptoms (Child Behaviour Checklist [CBCL] standard scores; Achenbach & Rescorla, 2000) and lower parent‐reported general communication skills (Children's Communication checklist‐Second Edition General Communication Composite; CCC‐2; Bishop, 2003) than their TD peers (all *p* < .001). The DSM‐orientated scales were also applied to the CBCL to derive the percentage of children above the clinical cut‐off for affective, anxiety and ADHD problems. See Table 1 for descriptive statistics and statistical tests of group differences. It is widely recognized that internalizing and externalizing symptoms are elevated in youth samples with ASD (Rosen, Mazefsky, Vasa, & Lerner, 2018). Indeed, the proportions of children with autism reaching criteria for anxiety (~40%) and ADHD symptoms (~30%) align with data from a recent study of children with autism utilizing the CBCL (Havdahl, Tetzchner, Huerta, Lord, & Bishop, 2016). The present sample appears to contain a slightly elevated proportion of children above the clinical cut‐off for affective symptoms (~40%, Haydahl et al reported ~20%), with average scores of 7.50 reported here.

**Table 1:** Descriptive statistics presented as *M*(*SD*) for demographic variables, cognitive measures and parent report questionnaires

	ASD (*n* = 20)	TD (*n* = 34)	*t/χ* ^2^	*d/w*
Age (months)	125.55 (16.09)	118.94 (17.59)	*t* = 1.41	*d* = 0.44
Gender (male:female)	16:4	17:17	*χ* ^2^3.59	*w* = 0.25
Cognitive measures				
BAS3 matrices	100.35 (19.76)	105.82 (16.96)	*t* = 1.03	*d* = 0.24
BAS3 word definitions	104.06 (19.15)	109.21 (13.64)	*t* = 1.02	*d* = 0.31
BPVS‐III	102.45 (14.04)	108.76 (12.02)	*t* = 1.68	*d* = 0.39
Parent questionnaires				
CSHQ total	52.87 (9.76)	40.94 (5.52)	*t* = 4.42***	*d* = 1.36
CBCL total	69.00 (7.15)	42.45 (8.93)	*t* = 11.40***	*d* = 3.14
% Affective	47.06	0.00		
% Anxiety	35.29	0.00		
% ADHD	29.41	0.00		
CCC−2 GCC	34.74 (15.10)	94.00 (10.07)	*t* = 15.26***	*d* = 3.91

Abbreviations: ASD, autism spectrum disorder; BAS3, British Ability Scales 3rd Edition; BPVS‐III, British Picture Vocabulary Scales, 3rd Edition; CBCL, Child Behaviour Checklist; CCC‐2 GCC, Children's Communication Checklist 2nd Edition General Communication Composite; CSHQ, Children's Sleep Habits Questionnaire; *d*/*w*, Cohen's *d*/*w* effect sizes; *t/χ* ^2^, independent *t* test/chi square.

* *p* ≤ .05;

** *p* ≤ .01; and

*** *p* ≤ .001.

Three participants in the ASD group were reported to be taking melatonin at the time of study intake (tablet: 4 mg and 9 mg and liquid: 4 ml). Regarding educational setting, one child in the TD group, and three children in the ASD group were home schooled. One child in the ASD group attended a school for children with social emotional and behavioural difficulties (SEBD). The remaining 91% of children attended mainstream schools and attended classes with their typically developing peers.

### 2.2 Stimuli

Nine mono‐ or bi‐syllabic rare words with 3 to 4 letters were selected (asp, goby, pipa, mata, uda, saki, gir, topi, paso). These were names of extant species/breeds of familiar animals (e.g., gir is a breed of cow), were judged to be unfamiliar to children aged 8–12 years, and were characterized by at least one unique physical feature (e.g.,a gir is a *humped* cow). They were allocated to a size category (small, medium or large) according to the rated size of their respective ‘base’ animal (e.g., cow) in existing norms (Paivio, 1975). Size categories were confirmed by data from 62 adults with animals rated on a scale from 1 (smallest) to 9 (largest). One 3‐letter and two 4‐letter words were chosen for each size category. A photograph of each novel animal was selected from Google images. In each, the animal took up approximately three‐fourth of the total photograph and all backgrounds were of a natural habitat. Nine familiar animal names were also selected for use in the size judgement task (worm, slug, rat, duck, goat, pig, cow, lion, bear). All were 3 or 4 letters in length with an Age of Acquisition (AoA) below 6 years (Kuperman, Stadthagen‐Gonzalez, & Brysbaert, 2012). The familiar animals were also allocated to a size group based on Paivio (1975) size norms. One 3‐letter and two 4‐letter words were chosen for each size category, based on those identified to be the most familiar to children aged 8–12 years (see Table S1 for stimuli lists).

### 2.3 Procedure

Participation involved three sessions. The *pre‐sleep* session consisted of the *training* tasks, followed by three tests of word learning in a fixed order (*size judgement, definitions task, naming speed*). The word learning tests were repeated again the following morning (*post‐sleep*), and again 1 month later (*follow‐up*). The pre‐sleep session began at approximately 6:30 p.m. (*M* = 6:27 p.m., *SD* = 0:39) and the post‐sleep session at approximately 9:30 a.m. The following morning (*M* = 9:23 a.m., *SD* = 0:34), following nocturnal sleep. Follow‐up took place approximately 1 month later (*M* = 32.07 days, *SD* = 5.52 days) at varying times across the day (*M* = 1:36p.m., *SD* = 3:12). Typically, the pre‐sleep session took place at home and the post‐sleep sessions in school. Whilst this could be viewed as a limitation of the design numerous studies have previously reported benefits of sleep on memory for newly learned material when the pre‐ and post‐sleep testing environments have been controlled; thus, it is unlikely that any sleep effects seen here could be attributed to the different testing environments. In all sessions, participants completed the *psychomotor vigilance task* (*PVT*) before any other tasks to measure alertness. In between pre‐ and post‐sleep sessions, participants underwent overnight home polysomnography. In a preliminary meeting, participants completed a battery of cognitive assessments including BAS word definitions and matrices subscales and the BPVS. Parents also completed the CSHQ, the CCC‐2, the CBCL and the GARS‐3.

Training and test sessions were delivered on DMDX (Forster & Forster, 2003) and the PVT task was administered using E‐prime experimental software (Psychology Software Tools).

#### 2.3.1 Training

Participants were told *Today you are going to learn some new words. All of the words are names for different types of animals. Some of the animals might look a bit like animals you already know*. Participants were then asked if they had heard of any of the animals before. Each novel animal name was presented via headphones and participants gave a yes/no verbal response. Yes responses were probed by the experimenter (‘Please describe a ____ to me?’).

Training consisted of 12 exposures to each novel word. In the first two exposures, participants heard the animal name and were asked to repeat it, after which the associated picture was presented onscreen for 3000 ms. In the following two exposures, participants saw the uppercase rare word onscreen and were asked to read the name out loud, after which the picture was presented for 3000 ms. Participants then completed a series of 2AFC trials with feedback. In image‐matching 2AFC trials, participants saw two images onscreen (one target and one distractor), to the left and right of the centre point. A novel written word was simultaneously presented centred underneath the images. Participants were asked to select, using a keypress, which image matched the word. Orthography‐matching trials were similar but with two words and one picture. The distractor was always another item from the stimulus set, with all items appearing an equal number of times throughout training. There was no timeout for this task and feedback was provided in the form of the target, which remained on screen for 2,000 ms. Participants completed four image‐matching, and four orthography‐matching trials, for each item in alternating blocks, with a different distractor for each exposure. Trial order was randomised within each block.

#### 2.3.2 Testing

##### Size judgement task

This task consisted of three blocks: familiar, mixed and novel. Familiar trials involved two familiar animals (e.g., BEE‐COW), mixed trials had one familiar and one novel animal (e.g., ASP‐COW or COW‐ASP) and novel trials contained only novel animals (e.g., GIR‐ASP). Twelve word pairs were selected for each block, six with a large semantic distance (large vs. small animal), and six with a small semantic distance (medium vs. small or medium vs. large). From all available pair combinations (27 per condition), pairs were selected based on letter length (matched where possible) and first letter (discrepant where possible). Each word pair was manipulated by screen location (x2) and congruency (×2), making four trials per pair (e.g., COW‐BEE, COW‐BEE, BEE‐COW, BEE‐COW), and a total of 48 trials in each block. Block order was counterbalanced between participants. Each trial consisted of two words typed in black uppercase Consolas font, spaced 40 mm apart against a white background. In congruent trials, the semantically larger animal word was 11 mm in height, and the semantically smaller animal word was 7 mm in height. In incongruent trials, the semantically smaller animal word was 11 mm in height, and the semantically larger animal word was 7 mm in height. Participants were instructed to decide which animal was largest in real life, as quickly and accurately as possible. Each trial began with a central fixation cross displayed for 600 ms, followed by the stimulus word pair presented either side of the central fixation cross. Participants used the laptop keyboard to respond as to whether the animal on the left (‘z’) or right (‘m’) was larger. Response timeout was set at 10,000 ms. Seven practice trials with feedback were completed prior to commencing the experimental blocks.

At the end of the follow‐up session only (to avoid influencing performance on other test), participants also completed a size‐ordering task. The purpose of this task was to check that participants’ perception of size aligned with the allocated small, medium and large categories. For the novel animals, participants were provided with nine cards, each with a picture of one novel animal. Participants were asked to order the animals from smallest (left) to largest (right). This therefore assessed participants’ perception of the size of the animals based solely on the trained image, as required for the size judgement task. For the familiar animals, participants were provided with the orthographic form (rather than an image) and again asked to order them from smallest (left) to largest (right). This part of the task therefore also served as a check for semantic knowledge of the familiar animals.

##### Definitions task

Each novel animal name was presented via headphones and participants were asked to describe the animal to the experimenter. Any responses which made reference only to the base animal (e.g., ‘a Gir is a cow’) were probed with a standard response of ‘can you tell me more about a ___?’ Separate scores were allocated for correctly recalling the ‘base animal’ and the feature. There was no timeout for this task.

##### Naming speed

Each novel animal picture was shown on screen and participants were asked to name the animal as quickly as possible. Timeout was set to 5,000 ms. Responses were recorded from picture onset via DMDX (Forster & Forster, 2003) and scored using CheckVocal (Protopapas, 2007) software. Accuracies and RTs were double‐scored and all discrepant accuracies and any RT differences >10 ms were checked and agreement was reached. One hundred per cent phonetic accuracy was required for each item to be scored as correct.

### 2.4 Sleep recordings

Home polysomnography recordings were completed using an ambulatory Embla titanium amplifier (Embla Systems Titanium) and RemLogic Version 3.4 software. Scalp sites were prepared with NuPrep exfoliating agent (Weave and Company) and electrodes were attached according to the international 10–20 system with a montage of six EEG (F3, F4, C3, C4, O1, O1), two EOG and two EMG channels. EEG and EOG channels were referenced offline to the contralateral mastoid and EMG channels were referenced to one another. Data were sampled at a rate of 256 Hz and EEG/EOG and EMG channels were bandpass filtered offline to 0.3–35 Hz and 10–100 Hz respectively.

Sleep stages were scored in accordance with Version 2.3 of the American Academy of Sleep Medicine (AASM; Berry et al., 2017) manual. All recordings were double‐scored by scorers who had extensive experience with scoring child sleep, with an average epoch‐by‐epoch concordance of 82.9%. Recordings were re‐coded in RemLogic allowing for blind scoring. Discrepancies greater than 10 consecutive 30‐s epochs (i.e., 5 min) were checked and agreement was reached. Prior to spindle and spectral analysis, artefacts were rejected manually using EEGLAB (Version 14.4.2). Spectral power analyses were conducted on artefact‐free NREM epochs using Fast Fourier Transformation on central channels (10–15 Hz). NREM spindles were detected and counted using an algorithm written by Tsanas and Clifford (2015), which uses a continuous wavelet transform (CWT) with a Morlet basis function to identify characteristic patterns of activity in central channels at 10–15 Hz. The first 3 hr of consecutive NREM sleep were included in the FFT and spindle analyses. This was done to maximize the number of usable datasets (with most data loss occurring in the second part of the night) and to allow us to capture roughly the first two sleep cycles where it has been argued that slow wave sleep is most prominent (at least in adults; Born, Rasch, & Gais, 2006). As such, participants were required to have at least 180 min of stages 2 and 3 sleep from a consecutive sample of EEG data from the time of sleep onset. Correlations between analyses based on the first 3 hr versus the whole night (where available) were nearly complete (C4 sigma power *r* = .95, C4 spindle density *r* = .96), validating our approach. To be included in the staging analyses, participants were required to have <10% unscored epochs across the night (*n* = 7 excluded on these grounds; four ASD and three TD). Reasons for unscored epochs included loss of Cz (upon which the titanium units depend) or removal of electrodes. Exploratory correlations were performed to assess the relationship between C3 and C4 spindle characteristics. Strong significant correlations were found between C3 and C4 sigma power [*r*(32) = .82, *p* < .001], density [*r*(32) = .99, *p* < .001] and duration [*r*(32) = .96, *p* < .001]. In accordance with AASM guidelines, C4 was selected as the dominant electrode and used for analysis. For participants with excessive artefacts in C4, C3 was used (ASD *n* = 3; TD *n* = 4).

### 2.5 Psychomotor Vigilance Task

To capture between‐group or between‐session baseline differences in alertness, participants completed a bespoke 90‐item psychomotor vigilance task (PVT) based on one developed by Basner and colleagues (Basner, Mollicone & Dinges, 2011). The task took approximately 4 min to complete. Participants were informed that a star would pop up on the screen intermittently and they were to click the mouse button as fast as they could. RT and frequency of lapses (RT > 500 ms) were recorded. ISIs ranged from 1,000 to 4,000 ms. There were no practice trials for this task.

### 2.6 Data analysis

All data were analysed in RStudio version 3.5 (RStudio Team, 2015) for R version 3.5.0 (R Core Team, 2018). Utilized packages include LME4 (Bates, Maechler, Bolker, & Walker, 2015), ggplot2 (Wickham, 2016) and emmeans (Lenth, 2019). Detailed analysis information can be found in the supplementary materials and data/scripts are freely available on the OSF: https://osf.io/bd9qy/?view_only=2e357aa59284476bb01860e94c15247f.

## 3 RESULTS

The following analysis presents a series of mixed effects regression models. For reference, unadjusted and untransformed participant‐level descriptive statistics for all tasks are shown in Table 2. Binomial GLMMs were used for accuracy data with response (0/1) as the DV and linear mixed effects models were used for RT data, with log‐transformed RT as the DV.

**Table 2:** Unadjusted group means for the language learning tasks, reported as *M* (*SD*)

	ASD		TD	
	Pre‐sleep	Post‐sleep	Pre‐sleep	Post‐sleep
Definitions				
Base (%)	69.3 (23.7)	77.1(18.6)	71.0 (16.9)	76.3 (18.8)
Feature (%)	53.6 (20.9)	64.1(17.8)	59.9 (21.0)	64.2 (19.2)
Naming				
Acc (%)	55.6 (26.1)	74.1 (25.0)	61.4 (25.1)	77.5 (20.2)
RT (ms)	1,984 (560)	1,775 (497)	1,891(594)	1,588 (437)
Size congruency				
Familiar RT (ms)	1,332 (426)	1,248 (618)	1,280 (346)	1,161 (331)
Mixed RT (ms)	2,010 (797)	1,550 (627)	1,923 (562)	1,533 (782)
Novel RT (ms)	2,058 (677)	1,479 (673)	2,194 (677)	1,509 (537)

### 3.1 Overnight consolidation

#### 3.1.1 Explicit memory (naming speed and definitions)

There was a significant overnight increase in naming accuracy, with item‐level responses more than three times (Session: OR = 3.13, *z* = 6.27, *p* < .001) as likely to be correctly recalled post‐sleep, relative to pre‐sleep. This overnight change was comparable between groups (Session*Group: OR = 1.16, *z* = 0.41, *p* = .67), and there was no significant difference between groups in overall task accuracy (Group: OR = 0.71, *z* = 0.76, *p *= .45). Similarly, there was a significant overnight decrease in RT (Session: *B* = −0.160, *z* = 4.90, *p *< .001) and this overnight decrease was comparable between groups (Session*Group: *B* = 0.018, *t* = 0.27, *p* = .78), with no significant difference between groups in overall task speed (Group: *B* = 0.092, *t* = 1.48, *p* = .15). Therefore, both groups demonstrated clear overnight improvements in phonological accuracy and retrieval speed. Table S2 shows the full model output.

There was also a significant yet notably smaller overnight increase in definitions accuracy for both base (OR = 1.59, *z* = 2.48, *p* = .013) and feature accuracy (OR = 1.47, *z* = 2.37, *p* = .018); correct responses were around 1.5 times more likely post‐sleep, relative to pre‐sleep. This overnight change was not significantly different between groups (Group*Session: OR_base_ = 1.21, *z* = 0.51, *p* = .61; OR_feature_ = 1.37, *z* = 0.96, *p* = .34) and overall task accuracy was comparable (Group: OR_base_ = 1.32, *z* = 0.08, *p* = .94; OR_feature_ = 0.85, *z* = 0.54, *p* = .59). Therefore, evidence was found for a pre‐sleep to post‐sleep improvement in expressive vocabulary performance, across both groups. See Table S3 for full model output.

#### 3.1.2 Semantic stabilization and integration (size judgement task)

Two TD participants and three ASD participants were excluded due to performance not significantly above chance (inclusion threshold ≥30 correct responses out of 48). One size congruency pair (MATA‐TOPI) was also removed from analyses due to low accuracy (*z* > 2.5; *M* = 68.3%). Forty‐five participants were therefore entered in to the RT model; 15 ASD and 30 TD. Performance accuracy was high across all blocks, with no significant difference between groups on familiar (ASD: 91.5% ± 5.5; TD: 92.0% ± 5.6; *t* = 0.32, *p* = .75), mixed (ASD: 90.5% ± 6.4; TD: 91.8% ± 6.2; *t* = 0.68, *p* = .50) or novel trials (ASD: 86.4% ± 7.0; TD: 89.0 ± 7.0; *t* = 1.18, *p* = .25). There was therefore sufficient evidence that participants understood the task demands.

##### Semantic stabilization

**Figure 1:**
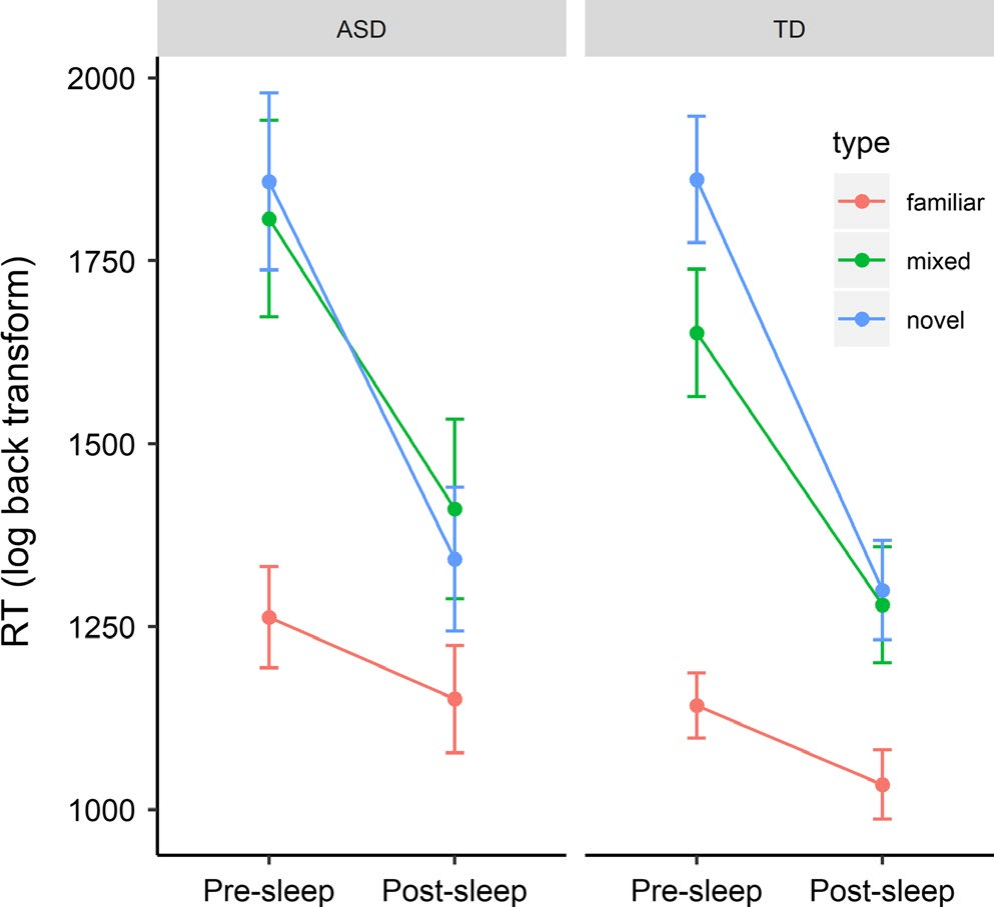
Estimated marginal means (adjusting for age) for size congruency RT as a function of block type, session and group. Error bars represent standard errors

Stabilization effects were explored first by fitting a model with the interaction terms for session, block and group. To recap, a significant session*type interaction indicates a pre‐sleep to post‐sleep RT change for novel/mixed trials that is distinct from familiar trials (i.e., overnight change controlling for practice effects). A session*type*group interaction indicates that these stabilization effects were different between groups. As previously stated, the role of age was explored in all models and was found to contribute significantly for this model, predicting overall RT; age was therefore retained as a fixed effect in the model. Significant session*type interactions (session*mixed: *B* = −0.16, *t* = 3.72, *p* < .001, Session*novel: *B* = −0.25, *t* = 7.90, *p* < .001) were identified and explored using emmeans. As shown in Figure 1 and Table 2, the pre‐sleep to post‐sleep decrease in RT was significantly greater for mixed (*z* = 6.68, *p* < .001) and novel (*z* = 13.37, *p* < .001) trials, relative to familiar trials (*z* = 3.00, *p* = .003). As such, post‐sleep task performance was characterized by more efficient semantic processing for items containing novel animals; suggesting overnight stabilization of the novel semantic information. Crucially, given that the model contrasts compared mixed and novel trials to familiar trials, these consolidation effects are unlikely to be a consequence of repeat test (i.e., practice) or circadian effects. That is, if practice or circadian confounds were responsible for these effects then they should also be influencing RTs to familiar trials. Synonymous with the definitions and naming task, this overnight consolidation was comparable between groups for novel (Session*novel*group: *B* = −0.03, *t* = 0.43, *p* = .67) and mixed (Session*novel*group: *B* < 0.01, *t* = 0.006, *p* = .99) trials. There was also a significant group*type interaction for novel trials relative to familiar. As shown in Figure 1, the ASD group showed less of an RT benefit for familiar animals relative to novel animals (*z* = 6.93, *p* < .001), compared to the TD group (*z* = 11.74, *p* < .001), perhaps as a consequence of less efficient processing of familiar animals. See Table S4 for full model output.

*Semantic integration*. Semantic integration effects were explored by assessing the roles of congruency and semantic distance. An overall semantic distance effect was observed (*B* = 0.11, *t* = 7.15, *p* < .001) with quicker RTs for trials with a large semantic distance than a small semantic distance; however, this effect was not consolidation‐dependent, with no relationship with session or block type (all *p* > .05). There was no significant effect of congruency, suggesting the absence of an overall congruency effect (*B* = 0.02, *t* = 1.68, *p* = .09). In exploratory analyses looking only at trials with a large semantic distance (following Tham et al., 2015), weak (and potentially spurious) congruency effects emerged in only two instances: (a) for familiar trials in the TD group in the pre‐sleep session (*z* = 2.30, *p* = .022), and (b) for novel trials in the ASD group in the pre‐sleep session (*z* = 2.54, *p* = .011).

#### 3.1.3 Month follow‐up

**Figure 2:**
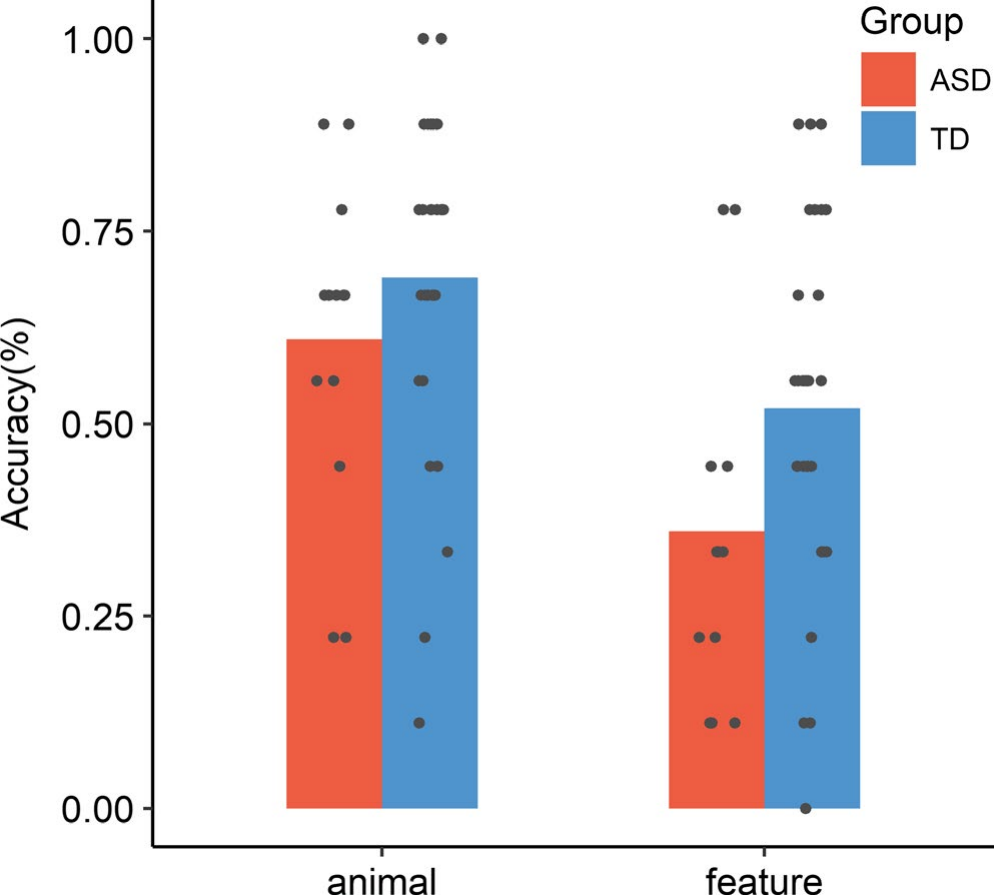
Participant‐level mean definitions accuracy at follow‐up; as a function of response type and group

Follow‐up data were available for 14 ASD and 32 TD participants. These subgroups were also matched on age (*t* = 1.41, *p* = .17), sex (*χ* ^2^ = 0.72, *p* = .39), receptive vocabulary (*t* = 1.68, *p* = .10), expressive vocabulary (*t* = 1.0, *p* = .32) and nonverbal ability (*t* = 1.04, *p* = .32). For the definitions task, the ASD group was comparable to TD children at recalling the base animals that were associated with the novel animals (e.g., asp is like a caterpillar; OR = 1.62, *z* = 1.22, *p* = .23), but they recalled significantly fewer unique features (e.g., asp is a *hairy* caterpillar; OR* = *2.24, *z* = 2.13, *p* = .033; Figure 2). Given that the groups were well matched for post‐sleep performance on definition of unique features (ASD: 64.1%, TD: 64.2%), this suggests that the TD children were better able to retain this knowledge over the subsequent month. Groups did not differ on naming accuracy (OR = 1.31, *z* = 0.51, *p* = .61) or speed (*B* < 0.01, *t* = 0.07, *p* = .95) or size judgement task speed (Group*mixed: *B* = 0.09, *t* = 1.52, *p* = .14, Group*novel: *B* = −0.01, *t* = 0.13, *p* = .90) at the month follow‐up.

### 3.2 Sleep characteristics

**Figure 3:**
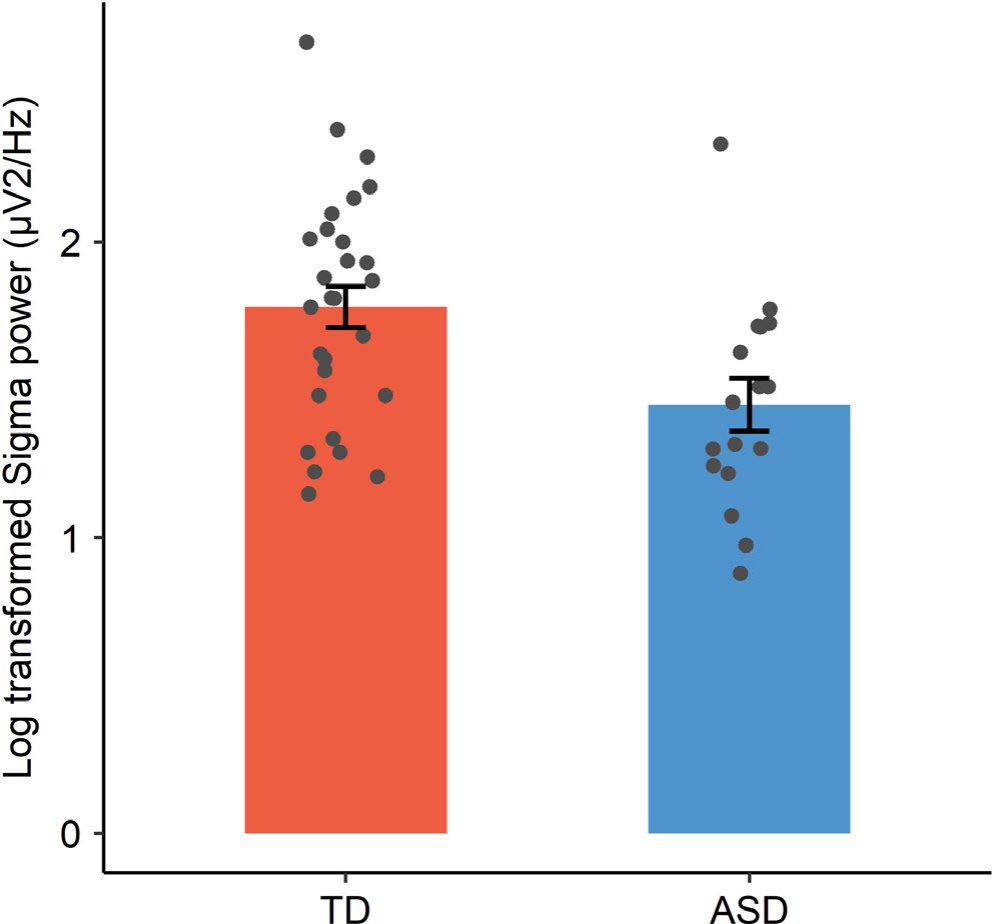
Mean log‐transformed sigma power for the TD and autism spectrum disorder groups. Error bars represent ±1 *SE* and points represent individual participants

Polysomnography data were available for 83.6% (17 ASD, 28 TD) of participants (see Table 3). Missing data were due to: (a) child opt‐out (ASD *n* = 1), (b) technical issues with the recording equipment (TD *n* = 3), (c) excessive artefact in both central channels (TD *n* = 3, ASD *n* = 2). The ASD group spent significantly less time in NREM sleep than the TD group (by 27 min; *p* = .028, *d* = 0.83). This result looked to be largely driven by 20 min less of N3, but there was no significant group difference in N3 duration in isolation, despite a medium effect size (*p* = .068, *d* = 0.67). No significant group differences were identified for spindle density (*p* = .97, *d* = 0.01) or spindle duration (*p* = .64, *d* = 0.09); however, the ASD group had significantly lower sigma power than the TD group, with a large effect size (*p* = .006, *d* = 0.89; Table 3 and Figure 3). Notably, this group difference in sigma power survives Bonferroni correction for multiple comparisons. Further exploration tentatively suggests that this group difference is driven primarily by power within the slow (10–12.5 Hz; *t* = 2.87, *p* = .006) but not fast (12.5–15 Hz; *t* = 1.47, *p* = .15) spindle frequency range. However, the fast spindle count was at floor for a number of children in both groups, and, more importantly, since no specific hypotheses were formed distinguishing fast versus slow spindles, these analyses should be viewed with caution and systematically tested in future research (note that fast/slow spindle data are available on the open science framework).

**Table 3:** Descriptive statistics presented as *M*(*SD*) for sleep variables

Sleep stages (min)	ASD (*n* = 13)	TD (*n* = 25)	*t*	*d*
TST	504.88 (36.03)	546.48 (37.11)	2.62*	1.14
N1 duration	19.85 (10.97)	26.46 (18.09)	1.40	0.44
NREM duration	361.54 (34.38)	387.76 (28.43)	2.36*	0.83
N2 duration	240.69 (46.26)	247.18 (39.91)	0.43	0.15
N3 duration	120.85 (31.27)	140.58 (27.77)	1.92^	0.67
REM duration	123.50 (27.52)	132.26 (30.40)	0.90	0.30
WASO	34.00 (34.40)	24.88 (16.64)	0.90	0.34

C4 Spindle characteristics	ASD (*n* = 17)	TD (*n* = 28)	*t*	*d*
Spindle density (per min)	4.17 (2.86)	4.20 (1.77)	0.04	0.01
Log‐transformed sigma power (10–15 Hz)	1.45 (0.35)	1.78 (0.39)	2.89**	0.89
Avg. spindle duration (s)	0.84 (0.12)	0.85 (0.10)	0.47	0.09

Abbreviations: ASD, autism spectrum disorder; TST, total sleep time; WASO, wake time after sleep onset.

^^^ *p* ≤ .1,

* *p* ≤ .05;

** *p* ≤ .01; and

There was a strong positive relationship between spindle density and spindle duration (*r*(43) = .83, *p* < .001), whereby children with more spindles on average per minute tended to have longer spindles (i.e., average duration in seconds per spindle). Furthermore, these two variables correlated with overall NREM sigma power (spindle density and sigma power; *r*(43) = .46, *p* = .002 and spindle duration and sigma power; *r*(43) = 0.55, *p* < .001), such that children with more NREM spindles and longer NREM spindles, also tended to have increased NREM sigma power. Age did not correlate with any sleep variable (all *r* < .3, *p* > .05).

### 3.3 The relationship between spindle characteristics and semantic stabilization

Given the lack of evidence for overnight changes in *semantic integration*, only the role of sleep in *semantic stabilization* was explored. As such, three sleep models were created, one for each sleep variable (sigma power, spindle duration, spindle density). As with the main model, block type contrasts compared mixed to familiar and novel to familiar trials. This reflects the notion that a role of sleep in semantic stabilization should be specific to trials containing novel animals, and not generalized to trials containing only familiar animals (i.e., with the latter reflecting an overall practice effect). The highest order interaction available was an interaction between the sleep variable and session*type*group, which captures the role of sleep in predicting overnight change in RT, across blocks and across group. To accompany each mode, Table 4 presents the *z* ratios for pre‐sleep to post‐sleep, for each block type and group. These *z* ratios are comparable to the more traditional correlations between sleep and overnight change. A positive *z* ratio indicates that the sleep variable predicted an overnight reduction in task speed (i.e., task improvement and support for our hypothesis) and a negative *z* ratio indicates that the sleep variable predicted an overnight increase in task speed. Task improvement is therefore synonymous with a positive *z* ratio.

**Table 4:** Z ratios for sleep characteristics predicting semantic judgement speed; pre‐sleep compared to post‐sleep

	ASD			TD		
	Power	Duration	Density	Power	Duration	Density
Familiar	3.37***	2.94**	4.45***	−2.50*	0.02	−0.02
Mixed	2.52*	2.95**	3.04**	−1.78	−0.58	−0.27
Novel	3.09**	1.11	1.59	1.94*	3.51***	−0.74

* *p* ≤ .05;

** *p* ≤ .01; and

*** *p* ≤ .001.

**Figure 4:**
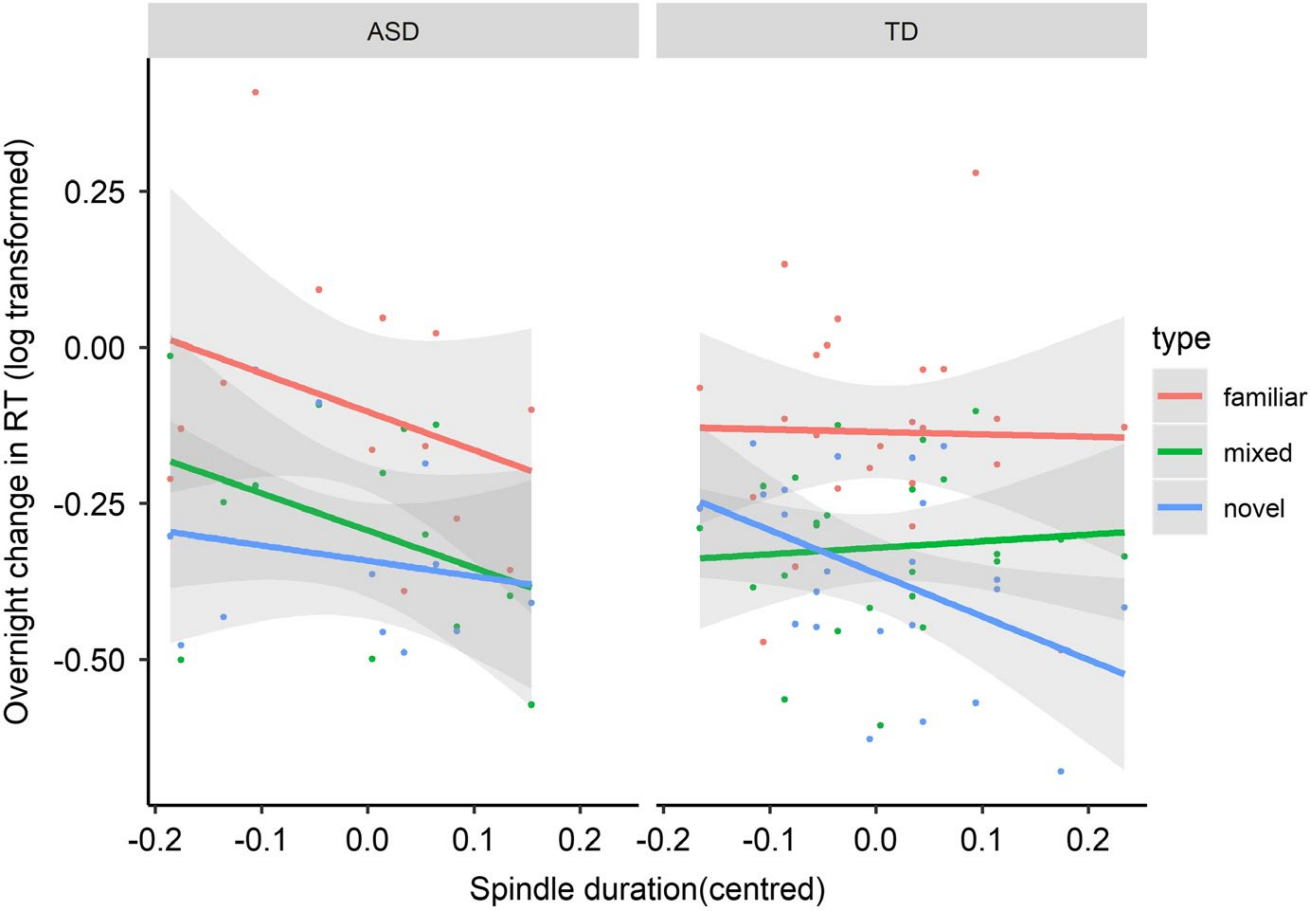
Spindle duration as a predictor of participant‐level overnight change in task RT. Points represent individual participants and shaded area represents 95% confidence interval for participant‐level regression line

For spindle duration, the highest order four‐way interaction was significant, specifically for the novel:familiar type contrast (*t* = 2.53, *p* = .011). As shown in Table 4 and Figure 4, this was accounted for by a direct dissociation in the role of spindle duration; predicting task improvement in *novel* trials for the TD group, but in *familiar* and *mixed* trials for the ASD group. Spindle density showed a significant three‐way interaction with session and group (*t = *4.79, *p* < .001), as did sigma power (*t = *5.21, *p* < .001). Namely, spindle density and sigma power predicted task improvement (collapsed across type) for the ASD group (density: *t* = 5.17, *p* < .001; power: *t* = 5.18, *p* < .001) but not the TD group (density: *t* = −0.61, *p* = .54; power: *t* = 1.25, *p* = .21). As shown in Table 4, this was characterized by these spindle properties predicting overnight semantic stabilization (i.e., task improvement) only for completely novel animal trials for the TD group. In fact, higher sigma power was associated with an overnight reduction in performance (i.e., slowing down) in familiar trials. In contrast, spindle density and sigma power predicted overnight task improvements more globally for children with ASD, working across familiar trials as well as trials containing novel animals. Notably, the associations were numerically strongest for the familiar words in the ASD group, where there was less to learn, at least semantically. To recall, though, the overall slower task speed in the ASD group for familiar trials relative to novel and mixed trials (supported by the group*type interaction shown in Figure 1) perhaps offered more opportunity for sleep to play a role in enhancing task performance for familiar trials. It is also important to note that when controlling for multiple comparisons using Bonferroni correction, the only *z* ratios to remain significant were for sigma power and spindle density predicting overnight change for familiar trials in the ASD group, and for spindle duration predicting overnight change for novel trials in the TD group. This bolsters our interpretation that the TD group are biased towards consolidating novel information, in contrast to the ASD group.

To summarize, associations between spindle properties and overnight semantic stabilization differed by group. For the TD group, spindle duration predicted overnight changes in responses to novel animals, but not changes in responses to familiar animals. In contrast, for children with ASD, sigma power and duration had a more holistic association with improvements in response speed for all types of trial, but particularly when trials contained familiar animals.

## 4 DISCUSSION

Sleep difficulties are commonly reported in childhood, particularly in neurodevelopmental disorders such as ASD. Despite this, little progress has been made in examining the impact of sleep on learning and development in these populations. In this endeavour, we examined the sleep‐associated consolidation of novel vocabulary in a relatively high ability, verbally able school‐aged children with ASD compared to TD peers matched on age, vocabulary and nonverbal ability. An assessment of sleep microstructure identified significantly lowers NREM central sigma activity in children with ASD, relative to TD peers. There was also some evidence of significantly reduced time in NREM sleep (mainly driven by reduced slow wave sleep duration in children with ASD). Nevertheless, children with and without ASD showed striking similarity in the extent to which they consolidated novel semantic knowledge overnight. More specifically, they showed equivalent performance when asked to define novel animals, name pictures of them, and make speeded semantic decisions about them immediately after learning, and both groups showed similar improvements after a single night of sleep. Spindle parameters predicted overnight improvements in speeded semantic judgements. Importantly, however, the nature of this relationship differed between groups. For the TD group, spindle parameters were specifically associated with performance on trials containing novel animals. Conversely, the associations were more general in the ASD group and strongest for trials containing already familiar animals, reflecting sleep‐associated improvements in task performance rather than with specific stabilization of *new* semantic knowledge. One month later, there was clear evidence that children with ASD were less likely to retain the unique (and defining) features of the novel animals. It is plausible, therefore, that the impact of sleep atypicalities and/or a lack of prioritization towards sleep‐dependent consolidation of new information in children with ASD may leave new semantic representations more vulnerable to the effects of long‐term forgetting.

### 4.1 Sleep characteristics in children with ASD

Mirroring numerous previous studies (e.g., Fletcher et al., 2017), parents of children with autism reported a higher rate of sleep problems than parents of typical peers. The CSHQ total scores were on average ~10 points higher in the autism group than in than the TD group.

The current work also demonstrates the feasibility of administering objective home‐based polysomnography in children with ASD. Whilst recruitment bias for this type of study is highly likely (and the present data do not reflect children with ASD who have more severe sensory, language and cognitive issues, for example), only one child with ASD chose not to wear the equipment, a number far lower than anticipated. Consistent with previous findings (Lambert et al., 2016) our data demonstrate that a sample of children with ASD who have language abilities within the normal range nevertheless have almost half an hour per night less of NREM sleep, mainly as a consequence of reduced SWS duration. Childhood is typically characterized by SWS‐rich sleep, with up to three times as long spent in SWS compared to adults (Wilhelm et al., 2013). The reduction of this in children with ASD may therefore have important implications for correlates of SWS, including declarative memory consolidation (Rasch & Born, 2013). It is important to note, however, that the group difference in NREM sleep did not survive correction for multiple comparisons, so should be interpreted with caution.

Regarding spindle characteristics, in line with Maski et al. (2015) and Lambert et al. (2016), we did not find evidence for reduced central spindle density in children with ASD (although note that Lambert et al., observed reduced *frontal* spindle density). Since previous studies more consistently report reduced spindle density in adults with ASD (Godbout et al., 2000; Limoges et al., 2005), and given findings that spindle density peaks around adolescence and reduces over adulthood (Purcell et al., 2017), it is possible that these findings reflect atypical maturation of spindle density in ASD.

Despite being well matched with TD peers on the prevalence of spindles in sleep and the typical duration of a spindle, the children with ASD showed significantly lower central NREM sigma power (i.e., the average power spectral density (PSD) within the spindle range, 10–15 Hz) supporting recent findings from Tessier et al. (2015). Thus, sleep spindles were lower in amplitude for children with ASD, compared to the amplitude of equivalent spindles in TD children. Although traditionally examined less often than spindle density, sigma power is gaining support as a robust predictor of general cognitive ability (e.g., Hoedlmoser et al., 2014; Tessier et al., 2015) and may be key to memory consolidation in developmental populations, as evidenced from studies of vocabulary consolidation (Smith et al., 2018) and nonverbal declarative memory (Maski et al., 2015). Evidence from neurotypical adults suggests that ‘spindle power’ (i.e., the average power of individually detected spindles, as opposed to the power within the sigma (spindle) frequency band, as measured here) reflects the structural integrity of an extensive network of white matter tracts including the forceps minor, parts of the uncinate fascicle and the anterior corpus callosum, as well as subcortical regions (including tracts within and around the thalamus; Piantoni et al., 2013). Thus, spindles reflect both the dynamics of network connectivity at the synaptic level (Poe, Walsh, & Bjorness, 2010; Tononi & Cirelli, 2006) as well as the state‐like network parameters that are governed by the structure of white matter tracts (Piantoni et al., 2013). Interestingly, reductions in white matter integrity, reflecting neocortical underconnectivity and local overconnectivity, are well documented from late childhood to adulthood in individuals with autism (e.g., Karahanoğlu et al., 2018). Further, it has been hypothesized that such disruptions to long‐range axonal projections in autism, crucial for the coordination of distributed neocortical activity, may impede cellular and systems consolidation (Runyan et al., 2019). Clearly, data are needed to fully characterize these differences to illuminate implications for learning and development.

### 4.2 Semantic learning and consolidation

Children with ASD and typical peers showed similar performance on the explicit measures of novel animal knowledge (i.e., naming speed and definitions accuracy) immediately after training, consistent with previous research (Henderson et al., 2014; Norbury et al., 2010). Furthermore, similar improvements were observed for these measures the following morning in both groups, again similar to Henderson et al.’s (2014) findings of intact overnight consolidation of novel word form knowledge in ASD. Thus, it appears that when learning via direct explicit instruction, children with ASD are akin to TD peers at encoding novel semantic information and consolidating these new memory traces overnight. Significant *improvements* in task performance, rather than maintenance, could signal an active role for sleep in supporting the consolidation of semantic knowledge in childhood, with sleep working to stabilize and integrate novel memories into existing semantic networks (Urbain et al., 2016). It is important to note, however, that the testing phase incorporated additional presentations of the novel animals which could have contributed to these offline improvements. For example, additional presentations could provide feedback for novel animals not accurately remembered in the initial tests (e.g., Krishnan, Sellars, Wood, Bishop, & Watkins, 2018). Nonetheless, overnight improvements in performance in similar word learning paradigms have been reported in the absence of repeat testing (e.g., Henderson, Weighall, & Gaskell, 2013). Furthermore, in studies where children are trained in the morning or evening and retested immediately, 12 hours and 24 hours later, improvements in recall are only observed at the 12 hour test for children trained in the evening (Henderson, Weighall, Brown, & Gaskell, 2012), implying that repeat testing cannot be solely responsible for overnight gains in performance.

There was, however, clear evidence that simple practice effects did not account for overnight changes in semantic stabilization. Namely, both groups of children showed significantly greater overnight reduction in overall semantic judgement RT for novel animals, than familiar animals. This is consistent with a consolidation effect that is specific to novel memory traces, as opposed to general practice effects on the task (similar to Tham et al., 2015). It should be noted, however, that children with ASD showed less of a differential consolidation benefit for novel versus familiar trials, with more of a tendency to also show a slight overnight improvement for familiar trials (Figure 1). This provides an initial suggestion that consolidation may be less strongly prioritized towards novel information in children with ASD (discussed further below, see Section 4.3).

The semantic judgement task was also included to capture semantic integration (i.e., as indexed by a size congruency effect). Counter to Tham et al. (2015), no clear post‐sleep congruency effects were observed for novel trials for typical peers or children with ASD. It is possible that the acquisition of novel semantic information may involve a more prolonged consolidation process in childhood in order to elicit congruency effects, which rely on automatic access to meaning upon written presentation of the word. Alternatively, the absence of these effects in children may more simply reflect increased variability in RTs relative to adults, rendering the congruency effect a less reliable marker of automatic semantic access in child populations. Consistent with this, there was only very weak evidence of a congruency effect even for familiar trials with a large semantic distance (e.g., COW ‐ BEE), which was confined to the typical peers.

Importantly, despite showing similar initial performance across all measures and evidence of overnight consolidation, children with ASD showed significantly greater rates of forgetting for the features of the novel animals roughly 1 month after training. This pattern of increased memory loss is strikingly similar to that of Norbury et al. (2010), where despite comparable performance on a definitions task shortly after learning, children with ASD recalled less semantic features of novel objects 1 month later in contrast to typical peers. This supports the notion of a prolonged consolidation process, whereby semantic information is gradually consolidated over a long period of time (McClelland et al., 1995). The fact that groups did not differ immediately after training or the next day suggests that the increased forgetting 1 month later cannot be a consequence of the pragmatic demands of this task (i.e., conversational strategy, prioritising relevance etc.). Instead, these data imply a more rapid decay of the integrity of semantic representations over time in ASD. This is consistent with previous reports that, in contrast to intact item memory, the ‘wheres’ and ‘whens’ of episodic memories are atypical in ASD, with generally poorer recall and reduced hippocampal connectivity during recall for such associations (Cooper et al., 2017; Cooper & Simons, 2018).

### 4.3 A role for sleep in semantic stabilization?

Spindles have been targeted as key to consolidation (e.g., Antony et al., 2018), and one previous study, to our knowledge, reports an association between overnight improvements in novel phonological knowledge and NREM spindle parameters in school‐aged children (Smith et al., 2018). Such data lend a developmental perspective to the predictions of the Complementary Learning Systems account of word learning (Davis & Gaskell, 2009), which proposes that this process engages two neural systems: the hippocampal system required for the rapid acquisition of a new word, and a slower learning neocortical system that enables strengthening of explicit knowledge as well as integration with existing vocabulary knowledge (Davis & Gaskell, 2009). The present data add to this evidence, showing that spindle parameters captured on the night after learning are also associated with overnight changes in the stabilization of novel *semantic* information. Specifically, we observed significant associations between sigma power and spindle duration and overnight change in semantic decision speed to novel animals, relative to familiar trials, in the typical children. The fact that these associations were specific to novel trials is crucial: This suggests that sleep is specifically targeting new memory traces, as opposed to general aspects of task performance.

Strikingly though, this same specificity of consolidation towards novel information was *not* observed for children with ASD. These children showed more general associations between sigma power and duration and overnight changes in semantic judgement RT to novel *and* familiar trials. In fact, only the associations between sigma power and density and overnight changes in semantic judgement RT to *familiar* trials survived correction for multiple comparisons in the ASD group. A similar pattern was reflected in the semantic judgement RT data, highlighted above, where children with ASD showed less of difference between familiar trials and trials containing novel animals pre‐ to post‐sleep. Together, these findings suggest that children with ASD may differ in how novel semantic information is prioritized for consolidation during sleep at the expense of other information encountered during the learning episode. This potential lack of prioritization *could* partly be a consequence of the integrity of their spindles (which are proposed as a fundament component of the architecture that supports the reactivation and consolidation of new memory traces; Staresina et al., 2015) and/or the quality of existing semantic representations. Regarding the latter explanation, semantic judgement RT to familiar trials was slower in ASD relative to typical peers. This resonates with previous findings of weaknesses in the flexibility with which semantic representations are retrieved during online language processing in autistic children with relatively good oral language skills (Henderson, Clarke, & Snowling, 2011; McCleery et al., 2010), lending further weight to the idea that long‐term semantic memories are more fragile in ASD.

### 4.4 Conclusions and implications

The current data add to an important body of evidence suggesting that sleep plays a role in language development, and that atypicalities of sleep may partly account for variability in language learning in neurodevelopmental disorders. Whilst the reasons for difficulties in the initiation of sleep are relatively well understood in ASD, the causal underpinnings of microstructural sleep atypicalities remain largely understudied. With clear evidence here that sigma power (and to a lesser extent NREM duration) are atypical even in a sample of children with ASD without co‐occurring language learning difficulties, causal factors for such profiles need to be explored in future research. Here, we have demonstrated that sleep spindles work to stabilize novel semantic memory traces in school‐aged children, with spindle characteristics specifically associated with overnight changes in novel (vs. familiar) material. In contrast, children with ASD showed reduced sigma power, more general associations between spindle characteristics and overnight changes in memory that were not prioritized towards the novel semantic information, and they showed greater forgetting of novel semantic features over the longer term. Thus, the behavioural consequences of reduced sigma power and/or general (vs. novel‐specific) consolidation processes may be most apparent after many iterations of the process (e.g., 1 month later), as opposed to just one (i.e., the following day).

Of course, the present findings apply only to one particular kind of semantic learning (i.e., the learning of rare but real animals) and only to a fraction of the autism population (i.e., without intellectual impairment and highly verbal individuals). Future research should aim to assess the generalizability of these findings across the spectrum and to the learning of other material. For instance, studies could address whether long‐term consolidation differs according to whether material is associated with a special interest. Notwithstanding these limitations, these data open up numerous theoretical and pedagogical questions, including how we might optimize consolidation in the autism population. For instance, repeated learning opportunities may be particularly beneficial for children with ASD, or modifying the training regimes to encourage prioritisation of the novel information to‐be‐learned.

## CONFLICT OF INTEREST

No conflict of interest exist for any authors.

## Data Availability

The datasets generated during and/or analysed during the current study are available on the OSF: https://osf.io/bd9qy/?view_only=2e357aa59284476bb01860e94c15247f.

## References

[desc12906-bib-0001] Achenbach, T. M. , & Rescorla, L. A. (2000). Manual for the ASEBA preschool forms & profiles. Burlington, VT: University of Vermont, Research Center for Children, Youth, & Families.

[desc12906-bib-0002] Antony, J. W. , Schönauer, M. , Staresina, B. P. , & Cairney, S. A. (2018). Sleep spindles and memory reprocessing. Trends in Neurosciences, 42(1), 1–3. 10.1016/j.tins.2018.09.012 30340875

[desc12906-bib-0003] Ashworth, A. , Hill, C. M. , Karmiloff‐Smith, A. , & Dimitriou, D. (2014). Sleep enhances memory consolidation in children. Journal of Sleep Research, 23(3), 304–310. 10.1111/jsr.12119 24329882

[desc12906-bib-0004] Ashworth, A. , Hill, C. M. , Karmiloff‐Smith, A. , & Dimitriou, D. (2017). A cross‐syndrome study of the differential effects of sleep on declarative memory consolidation in children with neurodevelopmental disorders. Developmental Science, 20(2), e12383 10.1111/desc.12383 PMC5347847 26690566

[desc12906-bib-0005] Baio, J. , Wiggins, L. , Christensen, D. L. , Maenner, M. J. , Daniels, J. , Warren, Z. , … Dowling, N. F. (2018). Prevalence of autism spectrum disorder among children aged 8 years – Autism and developmental disabilities monitoring network, 11 sites, United States, 2014. MMWR Surveillance Summary, 67(6), 1–23. 10.15585/mmwr.ss6706a1 PMC5919599 29701730

[desc12906-bib-0006] Basner, M. , Mollicone, D. , & Dinges, D. F. (2011). Validity and sensitivity of a brief psychomotor vigilance test (PVT-B) to total and partial sleep deprivation. Acta astronautica, 69(11–12), 949–959. 22025811 10.1016/j.actaastro.2011.07.015 PMC3197786

[desc12906-bib-0008] Bates, D. , Maechler, M. , Bolker, B. , & Walker, S. (2015). Fitting linear mixed‐effects models using lme4. Journal of Statistical Software, 67(1), 1–48.

[desc12906-bib-0009] Berry, R. B. , Brooks, R. , Gamaldo, C. , Harding, S. M. , Lloyd, R. M. , Quan, S. F. , … Vaughn, B. V. (2017). AASM scoring manual updates for 2017 (version 2.4). Journal of Clinical Sleep Medicine, 13(05), 665–666. 28416048 10.5664/jcsm.6576 PMC5406946

[desc12906-bib-0010] Bishop, D. V. (2003). The Children's Communication Checklist Version 2 (CCC-2). London, UK: Pearson.

[desc12906-bib-0011] Born, J. (2010). Slow-wave sleep and the consolidation of long-term memory. The World Journal of Biological Psychiatry, 11, 16–21. 20509828 10.3109/15622971003637637

[desc12906-bib-0012] Born, J. , Rasch, B. , & Gais, S. (2006). Sleep to remember. The Neuroscientist, 12(5), 410–424. 10.1177/1073858406292647 16957003

[desc12906-bib-0013] Cooper, R. A. , Richter, F. R. , Bays, P. M. , Plaisted‐Grant, K. C. , Baron‐Cohen, S. , & Simons, J. S. (2017). Reduced hippocampal functional connectivity during episodic memory retrieval in autism. Cerebral Cortex, 27(2), 888–902. 10.1093/cercor/bhw417 28057726 PMC5390398

[desc12906-bib-0014] Cooper, R. A. , & Simons, J. S. (2018). Exploring the neurocognitive basis of episodic recollection in autism. Psychonomic Bulletin & Review, 26(1), 163–181. 10.3758/s13423-018-1504-z PMC6424931 29987766

[desc12906-bib-0015] Crane, L. , Chester, J. W. , Goddard, L. , Henry, L. A. , & Hill, E. (2016). Experiences of autism diagnosis: A survey of over 1000 parents in the United Kingdom. Autism, 20(2), 153–162. 10.1177/1362361315573636 25810370

[desc12906-bib-0016] Davis, M. H. , & Gaskell, M. G. (2009). A complementary systems account of word learning: Neural and behavioural evidence. Philosophical Transactions of the Royal Society of London B: Biological Sciences, 364(1536), 3773–3800. 19933145 10.1098/rstb.2009.0111 PMC2846311

[desc12906-bib-0017] de Marchena, A. , Eigsti, I. M. , Worek, A. , Ono, K. E. , & Snedeker, J. (2011). Mutual exclusivity in autism spectrum disorders: Testing the pragmatic hypothesis. Cognition, 119(1), 96–113. 21238952 10.1016/j.cognition.2010.12.011

[desc12906-bib-0018] Diaz‐Roman, A. , Zhang, J. , Delorme, R. , Beggiato, A. , & Cortese, S. (2018). Sleep in youth with autism spectrum disorders: Systematic review and meta‐analysis. Evidence Based Mental Health, 21, 146–154. 30361331 10.1136/ebmental-2018-300037 PMC10270396

[desc12906-bib-0019] Diekelmann, S. , & Born, J. (2010). The memory function of sleep. Nature Reviews Neuroscience, 11(2), 114–126. 10.1038/nrn2762 20046194

[desc12906-bib-0020] Dunn, L. M. , Dunn, L. M. , & Styles, B. (2009). British Picture Vocabulary Scale (BPVS3) (3rd edn). London, UK: GL/Assessment.

[desc12906-bib-0021] Elliot, C. D. , & Smith, P. D. (2011). British Ability Scales (BAS-3) (3rd edn.). London, UK: GL/Assessment.

[desc12906-bib-0022] Fletcher, F. E. , Foster‐Owens, M. D. , Conduit, R. , Rinehart, N. J. , Riby, D. M. , & Cornish, K. M. (2017). The developmental trajectory of parent‐report and objective sleep profiles in autism spectrum disorder: Associations with anxiety and bedtime routines. Autism, 21(4), 493–503. 10.1177/1362361316653365 27354432

[desc12906-bib-0023] Forster, K. I. , & Forster, J. C. (2003). DMDX: A Windows display program with millisecond accuracy. Behavior Research Methods, Instruments, & Computers, 35(1), 116–124. 10.3758/bf03195503 12723786

[desc12906-bib-0024] Friedrich, M. , Mölle, M. , Friederici, A. D. , & Born, J. (2018). The reciprocal relation between sleep and memory in infancy: Memory‐dependent adjustment of sleep spindles and spindle‐dependent improvement of memories. Developmental Science, e12743. 30160012 10.1111/desc.12743 PMC6585722

[desc12906-bib-0025] Friedrich, M. , Wilhelm, I. , Born, J. , & Friederici, A. D. (2015). Generalization of word meanings during infant sleep. Nature Communications, 6, 6004 10.1038/ncomms7004 PMC4316748 25633407

[desc12906-bib-0026] Gais, S. , Lucas, B. , & Born, J. (2006). Sleep after learning aids memory recall. Learning & Memory, 13(3), 259–262. 16741280 10.1101/lm.132106 PMC10807868

[desc12906-bib-0027] Genzel, L. , Rossato, J. I. , Jacobse, J. , Grieves, R. M. , Spooner, P. A. , Battaglia, F. P. , … Morris, R. G. M. (2017). The yin and yang of memory consolidation: Hippocampal and neocortical. PLoS Biology, 15(1), e2000531 10.1371/journal.pbio.2000531 28085883 PMC5234779

[desc12906-bib-0028] Godbout, R. , Bergeron, C. , Limoges, E. , Stip, E. , & Mottron, L. (2000). A laboratory study of sleep in Asperger's syndrome. NeuroReport, 11(1), 127–130. 10683843 10.1097/00001756-200001170-00025

[desc12906-bib-0029] Gruber, R. , & Wise, M. S. (2016). Sleep spindle characteristics in children with neurodevelopmental disorders and their relation to cognition. Neural Plasticity, 2016, 4724792–4724792. 10.1155/2016/4724792 27478646 PMC4958463

[desc12906-bib-0030] Havdahl, K. A. , von Tetzchner, S. , Huerta, M. , Lord, C. , & Bishop, S. L. (2016). Utility of the child behavior checklist as a screener for autism spectrum disorder. Autism Research, 9(1), 33–42. 10.1002/aur.1515 26140652 PMC4939629

[desc12906-bib-0031] Henderson, L. M. , Clarke, P. J. , & Snowling, M. J. (2011). Accessing and selecting word meaning in autism spectrum disorder. Journal of Child Psychology and Psychiatry, 52(9), 964–973. 10.1111/j.1469-7610.2011.02393.x 21401594

[desc12906-bib-0032] Henderson, L. , Powell, A. , Gareth Gaskell, M. , & Norbury, C. (2014). Learning and consolidation of new spoken words in autism spectrum disorder. Developmental Science, 17(6), 858–871. 10.1111/desc.12169 24636285

[desc12906-bib-0033] Henderson, L. M. , Weighall, A. R. , Brown, H. , & Gareth Gaskell, M. (2012). Consolidation of vocabulary is associated with sleep in children. Developmental Science, 15(5), 674–687. 10.1111/j.1467-7687.2012.01172.x 22925515

[desc12906-bib-0034] Henderson, L. , Weighall, A. , & Gaskell, G. (2013). Learning new vocabulary during childhood: Effects of semantic training on lexical consolidation and integration. Journal of Experimental Child Psychology, 116(3), 572–592. 10.1016/j.jecp.2013.07.004 23981272

[desc12906-bib-0035] Hennies, N. , Ralph, M. A. L. , Kempkes, M. , Cousins, J. N. , & Lewis, P. A. (2016). Sleep spindle density predicts the effect of prior knowledge on memory consolidation. Journal of Neuroscience, 36(13), 3799–3810. 10.1523/JNEUROSCI.3162-15.2016 27030764 PMC4812136

[desc12906-bib-0036] Hoedlmoser, K. , Heib, D. P. , Roell, J. , Peigneux, P. , Sadeh, A. , Gruber, G. , & Schabus, M. (2014). Slow sleep spindle activity, declarative memory, and general cognitive abilities in children. Sleep, 37(9), 1501–1512. 10.5665/sleep.4000 25142558 PMC4153050

[desc12906-bib-0037] Hudry, K. , Leadbitter, K. , Temple, K. , Slonims, V. , McConachie, H. , Aldred, C. , … Pact Consortium (2010). Preschoolers with autism show greater impairment in receptive compared with expressive language abilities. International journal of language & communication disorders, 45(6), 681–690. 20102259 10.3109/13682820903461493

[desc12906-bib-0038] Hus, V. , Pickles, A. , Cook, E. H. Jr , Risi, S. , & Lord, C. (2007). Using the autism diagnostic interview—revised to increase phenotypic homogeneity in genetic studies of autism. Biological psychiatry, 61(4), 438–448. 17276746 10.1016/j.biopsych.2006.08.044

[desc12906-bib-0039] James, E. , Gaskell, M. G. , Weighall, A. , & Henderson, L. (2017). Consolidation of vocabulary during sleep: The rich get richer? Neuroscience & Biobehavioral Reviews, 77, 1–13. 28274725 10.1016/j.neubiorev.2017.01.054

[desc12906-bib-0040] Karahanoğlu, F. I. , Baran, B. , Nguyen, Q. T. H. , Meskaldji, D.‐E. , Yendiki, A. , Vangel, M. , … Manoach, D. S. (2018). Diffusion‐weighted imaging evidence of altered white matter development from late childhood to early adulthood in Autism Spectrum Disorder. NeuroImage: Clinical, 19, 840–847. 10.1016/j.nicl.2018.06.002 29946509 PMC6008282

[desc12906-bib-0041] Krishnan, S. , Sellars, E. , Wood, H. , Bishop, D. V. , & Watkins, K. E. (2018). The influence of evaluative right/wrong feedback on phonological and semantic processes in word learning. Royal Society Open Science, 5(9), 171496 10.1098/rsos.171496 30839710 PMC6170543

[desc12906-bib-0042] Kuperman, V. , Stadthagen‐Gonzalez, H. , & Brysbaert, M. (2012). Age‐of‐acquisition ratings for 30,000 English words. Behavior Research Methods, 44(4), 978–990. 10.3758/s13428-012-0210-4 22581493

[desc12906-bib-0043] Kurdziel, L. , Duclos, K. , & Spencer, R. M. (2013). Sleep spindles in midday naps enhance learning in preschool children. Proceedings of the National Academy of Sciences, 110(43), 17267–17272. 10.1073/pnas.1306418110 PMC3808582 24062429

[desc12906-bib-0044] Kurdziel, L. B. F. , & Spencer, R. M. C. (2016). Consolidation of novel word learning in native English‐speaking adults. Memory, 24(4), 471–481. 10.1080/09658211.2015.1019889 25768336 PMC4568164

[desc12906-bib-0045] Lambert, A. , Tessier, S. , Rochette, A.‐C. , Scherzer, P. , Mottron, L. , & Godbout, R. (2016). Poor sleep affects daytime functioning in typically developing and autistic children not complaining of sleep problems: A questionnaire‐based and polysomnographic study. Research in Autism Spectrum Disorders, 23, 94–106. 10.1016/j.rasd.2015.11.010

[desc12906-bib-0046] Latchoumane, C. F. V. , Ngo, H. V. V. , Born, J. , & Shin, H. S. (2017). Thalamic spindles promote memory formation during sleep through triple phase‐locking of cortical, thalamic, and hippocampal rhythms. Neuron, 95(2), 424–435. 10.1016/j.neuron.2017.06.025 28689981

[desc12906-bib-0047] Lenth, R. (2019). emmeans: Estimated marginal means, aka least‐squares means. R package version 1.3.2.

[desc12906-bib-0048] Limoges, E. , Mottron, L. , Bolduc, C. , Berthiaume, C. , & Godbout, R. (2005). Atypical sleep architecture and the autism phenotype. Brain, 128(Pt 5), 1049–1061. 10.1093/brain/awh425 15705609

[desc12906-bib-0049] Lo, B. H. , Klopper, F. , Barnes, E. H. , & Williams, K. (2017). Agreement between concern about autism spectrum disorder at the time of referral and diagnosis, and factors associated with agreement. Journal of Paediatrics and Child Health, 53(8), 742–748. 10.1111/jpc.13511 28374573

[desc12906-bib-0050] Luyster, R. , & Lord, C. (2009). Word learning in children with autism spectrum disorders. Developmental psychology, 45(6), 1774. 19899931 10.1037/a0016223 PMC3035482

[desc12906-bib-0051] Maski, K. , Holbrook, H. , Manoach, D. , Hanson, E. , Kapur, K. , & Stickgold, R. (2015). Sleep dependent memory consolidation in children with autism spectrum disorder. Sleep, 38(12), 1955–1963. 10.5665/sleep.5248 26194566 PMC4667378

[desc12906-bib-0052] McCleery, J. P. , Ceponienè, R. , Burner, K. M. , Townsend, J. , Kinnear, M. , & Schreibman, L. (2010). Neural correlates of verbal and nonverbal semantic integration in children with autism spectrum disorders. Journal of Child Psychology and Psychiatry, 51(3), 277–286. 10.1111/j.1469-7610.2009.02157.x 20025622

[desc12906-bib-0053] McClelland, J. L. , McNaughton, B. L. , & O'Reilly, R. C. (1995). Why there are complementary learning systems in the hippocampus and neocortex: Insights from the successes and failures of connectionist models of learning and memory. Psychological review, 102(3), 419. 7624455 10.1037/0033-295X.102.3.419

[desc12906-bib-0054] Muller, L. , Piantoni, G. , Koller, D. , Cash, S. S. , Halgren, E. , & Sejnowski, T. J. (2016). Rotating waves during human sleep spindles organize global patterns of activity that repeat precisely through the night. Elife, 5, e17267. 27855061 10.7554/eLife.17267 PMC5114016

[desc12906-bib-0055] Norbury, C. F. , Griffiths, H. , & Nation, K. (2010). Sound before meaning: Word learning in autistic disorders. Neuropsychologia, 48(14), 4012–4019. 10.1016/j.neuropsychologia.2010.10.015 20951710

[desc12906-bib-0056] Owens, J. A. , Spirito, A. , & McGuinn, M. (2000). The Children's Sleep Habits Questionnaire (CSHQ): Psychometric properties of a survey instrument for school‐aged children. Sleep, 23(8), 1043–1051. 10.1093/sleep/23.8.1d 11145319

[desc12906-bib-0057] Paivio, A. (1975). Perceptual comparisons through the mind’s eye. Memory & Cognition, 3(6), 635–647. 10.3758/BF03198229 24203905

[desc12906-bib-0058] Parish-Morris, J. , Hennon, E. A. , Hirsh-Pasek, K. , Golinkoff, R. M. , & Tager-Flusberg, H. (2007). Children with autism illuminate the role of social intention in word learning. Child development, 78(4), 1265–1287. 17650138 10.1111/j.1467-8624.2007.01065.x

[desc12906-bib-0059] Piantoni, G. , Poil, S. S. , Linkenkaer‐Hansen, K. , Verweij, I. M. , Ramautar, J. R. , Van Someren, E. J. , & Van Der Werf, Y. D. (2013). Individual differences in white matter diffusion affect sleep oscillations. Journal of Neuroscience, 33(1), 227–233. 10.1523/JNEUROSCI.2030-12.2013 23283336 PMC6618630

[desc12906-bib-0060] Plihal, W. , & Born, J. (1997). Effects of early and late nocturnal sleep on declarative and procedural memory. Journal of Cognitive Neuroscience, 9(4), 534–547. 10.1162/jocn.1997.9.4.534 23968216

[desc12906-bib-0061] Poe, G. R. , Walsh, C. M. , & Bjorness, T. E. (2010). Cognitive neuroscience of sleep. Progress in Brain Research, 185, 1–19. 21075230 10.1016/B978-0-444-53702-7.00001-4 PMC4180265

[desc12906-bib-0062] Prehn-Kristensen, A. , Munz, M. , Molzow, I. , Wilhelm, I. , Wiesner, C. D. , & Baving, L. (2013). Sleep promotes consolidation of emotional memory in healthy children but not in children with attention-deficit hyperactivity disorder. PLoS One, 8(5), e65098. 23734235 10.1371/journal.pone.0065098 PMC3667133

[desc12906-bib-0063] Preissler, M. A. (2008). Associative learning of pictures and words by low-functioning children with autism. Autism, 12(3), 231–248. 18445733 10.1177/1362361307088753

[desc12906-bib-0064] Protopapas, A. (2007). Check Vocal: A program to facilitate checking the accuracy and response time of vocal responses from DMDX. Behavior research methods, 39(4), 859–862. 18183901 10.3758/bf03192979

[desc12906-bib-0065] Purcell, S. M. , Manoach, D. S. , Demanuele, C. , Cade, B. E. , Mariani, S. , Cox, R. , … Stickgold, R. (2017). Characterizing sleep spindles in 11,630 individuals from the National Sleep Research Resource. Nature Communications, 8, 15930 10.1038/ncomms15930 PMC5490197 28649997

[desc12906-bib-0066] R Core Team . (2018). R: A language and environment for statistical computing. Vienna, Austria: R Foundation for Statistical Computing Retrieved from https://www.R-project.org/

[desc12906-bib-0067] RStudio Team . (2015). RStudio: Integrated development for R. Boston, MA: RStudio, Inc.

[desc12906-bib-0068] Rasch, B. , & Born, J. (2013). About sleep's role in memory. Physiological Reviews, 93(2), 681–766. 10.1152/physrev.00032.2012 23589831 PMC3768102

[desc12906-bib-0069] Rosanova, M. , & Ulrich, D. (2005). Pattern-specific associative long-term potentiation induced by a sleep spindle-related spike train. Journal of Neuroscience, 25(41), 9398–9405. 16221848 10.1523/JNEUROSCI.2149-05.2005 PMC6725710

[desc12906-bib-0070] Rosen, T. E. , Mazefsky, C. A. , Vasa, R. A. , & Lerner, M. D. (2018). Co‐occurring psychiatric conditions in autism spectrum disorder. International Review of Psychiatry, 30(1), 40–61. 10.1080/09540261.2018.1450229 29683351

[desc12906-bib-0071] Rubinsten, O. , & Henik, A. (2002). Is an ant larger than a lion? Acta Psychologica, 111(1), 141–154. 10.1016/S0001-6918(02)00047-1 12102118

[desc12906-bib-0072] Runyan, R. D. , Moore, A. N. , & Dash, P. K. (2019). Coordinating what we’ve learned about memory consolidation: Revisiting a unified theory. Neuroscience and Biobehavioural Reviews, 100, 77–84. 10.1016/j.neubiorev.2019.02.010 30790633

[desc12906-bib-0073] Smith, F. R. , Gaskell, M. G. , Weighall, A. R. , Warmington, M. , Reid, A. M. , & Henderson, L. M. (2018). Consolidation of vocabulary is associated with sleep in typically developing children, but not in children with dyslexia. Developmental Science, 21(5), e12639 10.1111/desc.12639 29226513

[desc12906-bib-0074] Smith, F. R. , & Henderson, L. M. (2016). Sleep problems in children with dyslexia: Understanding the role of sleep in neurocognitive development through the lens of developmental disorders. Acta Paediatrica, 105(9), 999–1000. 10.1111/apa.13506 27514003

[desc12906-bib-0075] Souders, M. C. , Mason, T. B. A. , Valladares, O. , Bucan, M. , Levy, S. E. , Mandell, D. S. , … Pinto‐Martin, J. (2009). Sleep behaviors and sleep quality in children with autism spectrum disorders. Sleep, 32(12), 1566–1578. 10.1093/sleep/32.12.1566 20041592 PMC2786040

[desc12906-bib-0076] Spanò, G. , Gómez, R. , Demara, B. , Cowen, S. , & Edgin, J. (2017). 0205 to nap or not to nap? sleep‐dependent memory consolidation in typically and atypically developing preschoolers. Sleep, 40(Suppl. 1), A76–A76. 10.1093/sleepj/zsx050.204

[desc12906-bib-0077] Staresina, B. P. , Bergmann, T. O. , Bonnefond, M. , van der Meij, R. , Jensen, O. , Deuker, L. , … Fell, J. (2015). Hierarchical nesting of slow oscillations, spindles and ripples in the human hippocampus during sleep. Nature Neuroscience, 18(11), 1679–1686. 10.1038/nn.4119 26389842 PMC4625581

[desc12906-bib-0078] Swensen, L. D. , Kelley, E. , Fein, D. , & Naigles, L. R. (2007). Processes of language acquisition in children with autism: Evidence from preferential looking. Child development, 78(2), 542–557. 17381789 10.1111/j.1467-8624.2007.01022.x

[desc12906-bib-0079] Tager-Flusberg, H. , Paul, R. , & Lord, C. (2005). Language and communication in autism. Handbook of autism and pervasive developmental disorders, 1, 335–364.

[desc12906-bib-0080] Tamminen, J. , Payne, J. D. , Stickgold, R. , Wamsley, E. J. , & Gaskell, M. G. (2010). Sleep spindle activity is associated with the integration of new memories and existing knowledge. Journal of Neuroscience, 30(43), 14356–14360. 10.1523/JNEUROSCI.3028-10.2010 20980591 PMC2989532

[desc12906-bib-0081] Tessier, S. , Lambert, A. , Chicoine, M. , Scherzer, P. , Soulières, I. , & Godbout, R. (2015). Intelligence measures and stage 2 sleep in typically‐developing and autistic children. International Journal of Psychophysiology, 97(1), 58–65. 10.1016/j.ijpsycho.2015.05.003 25958790

[desc12906-bib-0082] Tham, E. K. H. , Lindsay, S. , & Gaskell, M. G. (2015). Markers of automaticity in sleep‐associated consolidation of novel words. Neuropsychologia, 71, 146–157. 10.1016/j.neuropsychologia.2015.03.025 25817848

[desc12906-bib-0083] Tononi, G. , & Cirelli, C. (2006). Sleep function and synaptic homeostasis. Sleep Medicine Reviews, 10, 49–62. 10.1016/j.smrv.2005.05.002 16376591

[desc12906-bib-0084] Tsanas, A. , & Clifford, G. D. (2015). Stage‐independent, single lead EEG sleep spindle detection using the continuous wavelet transform and local weighted smoothing. Frontiers in Human Neuroscience, 9, 181 10.3389/fnhum.2015.00181 25926784 PMC4396195

[desc12906-bib-0085] Tucker, M. A. , Hirota, Y. , Wamsley, E. J. , Lau, H. , Chaklader, A. , & Fishbein, W. (2006). A daytime nap containing solely non‐REM sleep enhances declarative but not procedural memory. Neurobiology of Learning and Memory, 86(2), 241–247. 10.1016/j.nlm.2006.03.005 16647282

[desc12906-bib-0086] Urbain, C. , De Tiège, X. , Op De Beeck, M. , Bourguignon, M. , Wens, V. , Verheulpen, D. , … Peigneux, P. (2016). Sleep in children triggers rapid reorganization of memory‐related brain processes. NeuroImage, 134, 213–222. 10.1016/j.neuroimage.2016.03.055 27039143

[desc12906-bib-0087] Weighall, A. , Henderson, L.‐M. , Barr, D. , Cairney, S. A. , & Gaskell, M. G. (2017). Eye‐tracking the time‐course of novel word learning and lexical competition in adults and children. Brain and Language, 167, 13–27. 10.1016/j.bandl.2016.07.010 27562102

[desc12906-bib-0088] Wickham, H. (2016). ggplot2: elegant graphics for data analysis. New York: Springer‐Verlag.

[desc12906-bib-0089] Wilhelm, I. , Diekelmann, S. , & Born, J. (2008). Sleep in children improves memory performance on declarative but not procedural tasks. Learn Mem, 15(5), 373–377. 10.1101/lm.803708 18441295

[desc12906-bib-0090] Wilhelm, I. , Rose, M. , Imhof, K. I. , Rasch, B. , Büchel, C. , & Born, J. (2013). The sleeping child outplays the adult's capacity to convert implicit into explicit knowledge. Nature Neuroscience, 16(4), 391–393. 10.1038/nn.3343 23434910

[desc12906-bib-0091] Williams, S. E. , & Horst, J. S. (2014). Goodnight book: Sleep consolidation improves word learning via storybooks. Frontiers in Psychology, 5, 184 10.3389/fpsyg.2014.00184 24624111 PMC3941071

