# Supplementary material for: Atypicalities in sleep and semantic consolidation in autism
Source: Dev Sci. 2019 Oct 20;23(3):e12906. doi: 10.1111/desc.12906 (PMC7187235; doi:10.1111/desc.12906)
Supplement: Supplementary file 1 [file DESC-23-e12906-s001.docx]

Supplementary Method and Results

Table S1

*Stimuli: Familiar and novel animal names*

| **Size category** | **Familiar animal (size rating)** | **Novel  animal name** | **Novel animal base (size rating)** | **Novel animal feature** |
| --- | --- | --- | --- | --- |
| Small | worm (*M* = 1.45, *SD* = 0.61) | asp | caterpillar; *M* = 1.50, *SD* = 0.62 | furry |
|  | slug (*M* = 1.47, *SD* = 0.59) | goby | goldfish; *M* = 1.85, *SD* = 0.59 | see through/translucent |
|  | rat (*M* = 1.86, *SD* = 0.69) | pipa | frog; *M* = 2.26, *SD* = 0.65 | flat |
| Medium | duck (*M* = 3.24, *SD* = 0.80) | mata | turtle; *M* = 3.48, *SD* = 1.13 | long neck |
|  | goat (*M* = 4.76, *SD* = 0.95) | uda | sheep; *M* = 4.81, *SD* = 0.90 | half black/half white |
|  | pig (*M* = 4.84, *SD* = 0.91) | saki | monkey; *M* = 4.56, *SD* = 0.75 | bright white/yellow face |
| Large | cow (*M* = 6.71, *SD* = 1.02) | gir | cow; *M* = 6.71, *SD* = 1.02 | humped back |
|  | lion (*M* = 6.87, *SD* = 0.93) | topi | antelope; *M* = 6.05, *SD* = 1.33 | yellow stripy legs |
|  | bear (*M* = 7.55, *SD* = 0.96) | paso | horse; *M* = 7.13, *SD* = 0.99 | long curly mane/hair |

Table S2

*Predictors of naming task accuracy and RT (n=52)*

|  |  | *Fixed effects* | | | | |  | *Random effects (SD)* | |
| --- | --- | --- | --- | --- | --- | --- | --- | --- | --- |
|  |  | *B* | *SE* | *CI* | | *z* |  | *Code* | *item* |
| DV | Predictor |  |  | *2.5%* | *97.5%* |  |  |  |  |
| Accuracy | **Intercept** | **1.10** | **0.37** | **0.38** | **1.83** | **2.97**** |  | 1.39 | 0.89 |
|  | **Session** | **1.14** | **0.18** | **0.78** | **1.50** | **6.27***** |  | - | - |
|  | Group | -0.34 | 0.44 | -1.20 | 0.53 | 0.76 |  | - | - |
|  | Session:group | 0.15 | 0.36 | -0.55 | 0.85 | 0.41 |  | - | - |
| RT | **(Intercept)** | **7.399** | **0.047** | **7.306** | **7.492** | **156.01***** |  | 0.18 | 0.11 |
|  | **Session** | **-0.160** | **0.033** | **-0.225** | **-0.096** | **4.90***** |  | - | - |
|  | Group | 0.092 | 0.062 | -0.030 | 0.214 | 1.48 |  | - | - |
|  | Session:Group | 0.018 | 0.065 | -0.110 | 0.146 | 0.27 |  | - | - |

† Models formed from 936 observations: 52 participants across 9 items and 2 sessions.  **p*⩽0.05; ***p*⩽0.01; and ****p*⩽0.001.

Table S3

*Predictors of base and feature definitions task accuracy (n=48)*

|  |  | *Fixed effects* | | | | |  | *Random effects (SD)* | |
| --- | --- | --- | --- | --- | --- | --- | --- | --- | --- |
|  |  | *B* | *SE* | *CI* | | *z* |  | *Code* | *item* |
| DV | Predictor |  |  | *2.5%* | *97.5%* |  |  |  |  |
| Definitions base | **Intercept** | **1.47** | **0.41** | **0.66** | **2.27** | **3.58***** |  | 1.05 | 1.08 |
|  | **Session** | **0.46** | **0.19** | **0.10** | **0.83** | **2.48*** |  | - | - |
|  | Group | 0.03 | 0.37 | -0.70 | 0.76 | 0.08 |  | - | - |
|  | Session:group | 0.19 | 0.37 | -0.54 | 0.92 | 0.51 |  | - | - |
| Definitions featur**e** | Intercept | 0.52 | 0.29 | -0.05 | 1.10 | 1.78 |  | 0.81 | 0.76 |
|  | **Session** | **0.38** | **0.16** | **0.07** | **0.70** | **2.37*** |  | - | - |
|  | Group | -0.16 | 0.30 | -0.74 | 0.42 | 0.54 |  | - | - |
|  | Session:group | 0.31 | 0.32 | -0.32 | 0.95 | 0.96 |  | - | - |

† Models formed from 864 observations: 48 participants across 9 items and 2 sessions.  **p*⩽0.05; ***p*⩽0.01; and ****p*⩽0.001.

Table S4

*Predictors of logRT size congruency speed (n=45)*

| Predictor | *Fixed effects* | | | | |  | *Random effects (SD)* | |
| --- | --- | --- | --- | --- | --- | --- | --- | --- |
|  |  |  | *CI* | |  |  |  |  |
|  | *B* | *SE* | *2.5%* | *97.5%* | *t* |  | *code* | *item* |
| Intercept | **7.24** | **0.04** | **7.17** | **7.31** | **197.97***** |  | 0.22 | 0.09 |
| **Session1** | **-0.23** | **0.02** | **-0.28** | **-0.18** | **9.82***** |  | 0.14 | - |
| **type(mixed)** | **0.29** | **0.03** | **0.22** | **0.35** | **8.30***** |  | 0.18 | - |
| **type(novel)** | **0.31** | **0.03** | **0.26** | **0.37** | **11.19***** |  | 0.12 | - |
| Group | 0.07 | 0.07 | -0.07 | 0.22 | 0.96 |  | - | - |
| **age** | **-0.14** | **0.03** | **-0.20** | **-0.09** | **4.89***** |  | - | - |
| **Session:type(mixed)** | **-0.16** | **0.04** | **-0.24** | **-0.07** | **3.72***** |  | 0.24 | - |
| **Session:type(novel)** | **-0.25** | **0.03** | **-0.31** | **-0.18** | **7.90***** |  | 0.16 | - |
| Session:Group | 0.02 | 0.05 | -0.08 | 0.11 | 0.34 |  | - | - |
| type(mixed):Group | -0.01 | 0.06 | -0.12 | 0.10 | 0.16 |  | - | - |
| **type(novel):Group** | **-0.09** | **0.04** | **-0.17** | **-0.01** | **2.11*** |  | **-** | **-** |
| Session:type(mixed):Group | 0.00 | 0.08 | -0.16 | 0.16 | 0.01 |  | - | - |
| Session:type(novel):Group | 0.03 | 0.06 | -0.10 | 0.15 | 0.43 |  | - | - |
|  |  |  |  |  |  |  |  |  |

†Model formed from 11384 obs; 45 participants across 140 items. *p⩽0.05; **p⩽0.01; and ***p⩽0.001. ‘novel’ refers to the novel-familiar contrast and ‘mixed’ refers to the mixed-familiar contrast.

**Supplementary method – Data analysis**

***Group-level analyses***

PVT lapses, Sigma power and spindle density were not normally distributed, and were therefore log transformed prior to analyses. Independent sample t-tests were then performed to assess group differences. Group differences in gender were assessed with a chi-squared test for independence. Reported effect sizes include Cohen’s *d* (values ⩾0.2/⩾0.5/⩾0.8=small/medium/large) and *W* for chi squared (values ⩾0.1/⩾0.3/⩾0.5=small/medium/large).

***Mixed-effects (item level) analyses***

One ASD participant chose not to complete any testing tasks upon the completion of the training tasks, and another ASD participant did not complete any training or test tasks, but both completed the overnight polysomnography recording and were therefore retained for the sleep analyses only. In addition, one ASD participant completed all testing tasks except the definitions task, due to time constraints on the evening of testing. A further three TD participants had missing definitions data due to lost datasheets and two TD participants had missing post-sleep size congruency data due to technical issues with task administration. As such, data were available for 48 participants for the definitions task (ASD *n*=17;TD *n*=31), 52 participants for the naming task (ASD *n*=18;TD *n*=34), and 50 participants for the SC task (ASD *n*=18; TD *n*=32). Fixed effects were coded using simple contrasts, with all models including fixed effects of session (coding:pre-sleep=-0.5, post-sleep=+.5) and group (coding:TD=-0.5, ASD=+.5). For the size congruency task, additional fixed effects of block type (familiar-mixed-novel; contrast 1:familiar=-1/3, mixed=+2/3, novel=-1/3, contrast 2:familiar=-1/3, mixed=-1/3  novel=+2/3) were included. Contrast 1 represented speed to mixed trials relative to familiar, and contrast 2 represents speed to novel trials relative to familiar. Congruency was also included in the size congruency model (congruent=-0.5, incongruent=+.5). Prior to entry in the size congruency model, item-level RTs less than 300 ms were removed from analyses (99.40% of trials retained). For each model, by-participant and by-item slopes were included if justified by improved model fit under a liberal criterion of *p*<.2, via likelihood ratio tests (Bates, Kliegl, Vasishth, & Baayen, 2015). Slopes for the intercept with the largest variance (typically participant) were built up first. The slope with the smallest *p*-value in model comparison (i.e., the slope which showed the greatest model improvement) was retained, with the process repeated until no further model improvement (i.e., *p*<.2) could be achieved. The statistical significance of each fixed effect was determined using the Satterthwaite approximation from the lmerTest package (Kuznetsova et al., 2017), which has been shown to provide a more conservative estimate than likelihood ratio tests, particularly for small sample sizes (Luke, 2017).

In the final model, influential cases were assessed by examination of dfbetas using the influence.me package (Nieuwenhuis, 2017). Dfbetas provide an estimate of each participant’s influence on the beta coefficient of each fixed effect. dfbetas were standardised and influential cases were classified as cases with a z score greater than +/- 3.29. The model was checked for robustness by performing leave-one-out model refits for each influential case. Unless otherwise specified, all models were robust to outliers and met the assumption of normality of residuals. 95% CIs for all fixed effects were provided using Wald method in lme4. Binomial GLMM’s were used for accuracy data with item-level accuracy as the DV. For interpretability, beta coefficients are converted to odds ratios (OR; i.e., exp(B)). LME models were fitted to RT data for correct responses only, on log transformed RTs.

To assess the role of sleep, the three spindle variables were centred and scaled, and added in to each model. To avoid issues with multicollinearity, separate models were created for each sleep variable (i.e., sigma power, spindle duration and spindle density). Whilst summary model parameters are reported in text, all full model outputs can be found (including random effect structures) in the supplementary materials. The role of age was assessed for all variables, unless otherwise specified, age did not predict task outcome.

Supplementary measures

**1) Prior knowledge check**

In response to the ‘do you know this animal’ question (administered at the start of the training), 95.5% of responses indicated no prior knowledge of the animal name. 2.9% of responses indicated prior knowledge but when probed with “can you describe a ____ to me?” participants could not provide a valid description. Of the remaining 1.6% of responses, 1 participant provided a correct response ‘fish’ when asked to describe a goby, and 1 participant correctly described a paso as a ‘horse.’ In addition, 4 participants (3 TD and 1 ASD) described an asp as a snake.

**2) Size ordering**

Size ordering data were available for 33 of the 45 children included in the main SC task analyses. Accuracy for the familiar animals was 100%, such that all participants allocated the small (worm, slug, rat), medium (duck, pig, goat), and large (cow, lion, bear) animals to the correct size category. This therefore confirmed both semantic knowledge of the animals, and correct size knowledge. For the novel animals, 73.5% of children allocated all animals to the correct size category. Of the 9 children (26.5%) with incorrect allocations (TD n=8, ASD n=1), 7 of these were characterised by incorrect allocation of topi and/or mata(medium instead of large). This explains the low accuracy of the MATA-TOPI item. As stated previously, the MATA-TOPI items were removed from analyses, and as such, these incorrect size judgements should have no influence on the included size congruency analyses.

**3) PVT**

There was no significant difference in the number of lapses from pre-sleep to post-sleep (F (50) = 0.59, p >. 05), indicating an absence of confounding circadian effects. There were also no significant differences between groups in overall number of lapses (F (50) = 3.46, p >. 05) or the change in lapses from pre-sleep to post sleep (Group*Session: F (50) = 3.32 p >. 05); indicating an absence of confounding effects of group level attention or fatigue on task performance. At month follow up (T3), there was also no significant group difference in the number of lapses (t (39) = 0.73, p >0.05).
